# Synthesis of a Hexachloro Sulfate(IV) Dianion Enabled by Polychloride Chemistry

**DOI:** 10.1002/anie.202209684

**Published:** 2022-08-30

**Authors:** Patrick Voßnacker, Alisa Wüst, Carsten Müller, Merlin Kleoff, Sebastian Riedel

**Affiliations:** ^1^ Institut für Chemie und Biochemie—Anorganische Chemie Freie Universität Berlin Fabeckstraße 34/36 14195 Berlin Germany

**Keywords:** Ionic Liquids, Polychlorides, Quantum Chemistry, Sulfur, VSEPR Model

## Abstract

The preparation and structural characterization of [NEt_3_Me]_2_[SCl_6_] is described, which is the first example of a [SCl_6_]^2−^ dianion and of a halosulfate anion of the type [S_*x*_X_*y*_]^*z*−^ in general. This dianion belongs to the group of 14‐valence electron AB_6_E systems and forms an octahedral structure in the solid‐state. Interestingly, co‐crystallization with CH_2_Cl_2_ affords [NEt_3_Me]_2_[SCl_6_]⋅4 CH_2_Cl_2_ containing [SCl_6_]^2−^ dianions with *c*_4*v*_ symmetry. As suggested by quantum‐chemical calculations, the distortion of the structure is not caused by a stereochemically active lone pair but by enhanced hydrogen bonding interactions with CH_2_Cl_2_. At elevated temperatures, [NEt_3_Me]_2_[SCl_6_] decomposes to various sulfur chlorine compounds as shown by Raman spectroscopy. Cooling back to room temperature results in the selective formation of [NEt_3_Me]_2_[SCl_6_] which is comparable to the well‐studied SCl_4_.

## Graphical abstract

The first preparation and structural characterization of the hexachlorosulfate dianion, [NEt_3_Me]_2_[SCl_6_], is achieved. This synthesis is enabled by the Janus‐faced trichloride reagent [NEt_3_Me][Cl_3_]. While the trichloride [Cl_3_]^−^ is chlorinating and oxidizing elemental sulfur, the unique [NEt_3_Me]^+^ cation is stabilizing the unprecedented [SCl_6_]^2−^ dianion.

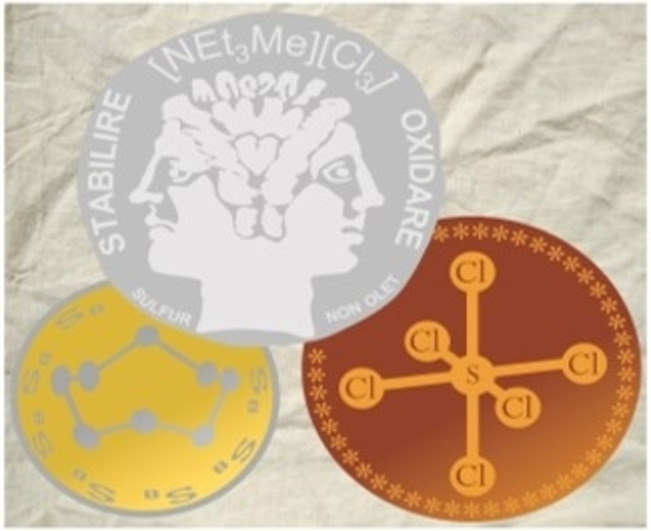

The chlorination of small molecules is a topic of major relevance both for industrial and academic research.^[1]^ Very recently, our group in cooperation with Covestro investigated [NEt_3_Me]Cl as a practical chlorine storage medium which forms the corresponding trichloride [NEt_3_Me][Cl_3_] by addition of chlorine.^[2]^ More importantly, we found that [NEt_3_Me][Cl_3_] is also a useful chlorinating agent reacting with carbon monoxide to the base chemical phosgene (COCl_2_). Interestingly, our studies indicate that the trichloride anion in [NEt_3_Me][Cl_3_] has a Cl−Cl bond which is much weaker than in elemental chlorine facilitating the insertion of carbon monoxide into the Cl−Cl bond of the trichloride.^[3]^

In contrast to elemental chlorine, [NEt_3_Me][Cl_3_] contains the chemically inert cation [NEt_3_Me]^+^ which has proven to be very useful to stabilize various reactive anions.^[4]^ Therefore, we speculated that the exceptional ability of [NEt_3_Me][Cl_3_] to serve both as a strong chlorination reagent and to form stable salts with unique anions would enable the preparation of unknown chlorine‐containing compounds. While focusing on sulfur compounds, we noted that most sulfur chlorides are relatively unstable species. Reaction of elemental sulfur with chlorine leads primarily to S_2_Cl_2_, which can be further reacted with an excess of chlorine in the presence of FeCl_3_ as catalyst to SCl_2_.^[5]^ While S_2_Cl_2_ is comparably stable, SCl_2_ decomposes at room temperature slowly to S_2_Cl_2_ and Cl_2_. At −78 °C, SCl_2_ reacts with liquid Cl_2_ to SCl_4_ but decomposes to SCl_2_ and Cl_2_ when warmed above its melting point of −30 °C. Below that temperature, SCl_4_ exists in the ionic structure [SCl_3_]^+^[Cl]^−^, as suggested by IR and Raman spectroscopy and powder XRD analysis.^[6]^ Beside SCl_4_, various salts of the type [SCl_3_]^+^[X]^−^ with, e.g., X=[ICl_4_],^[7]^ [SbF_6_],^[8]^ [F(Al(OC_4_F_9_)_3_)_2_]^[9]^ have been prepared. The highest possible binary sulfur chlorine species, SCl_6_, is not known, while the lighter homologue SF_6_ is a stable compound that found various industrial applications.^[10]^ Surprisingly, chlorosulfates, [S_*x*_Cl_*y*_]^*z*−^, have not been prepared thus far, although afterglow‐tandem mass spectrometric experiments gave a *D*_0_(SCl_2_−Cl^−^) bond energy of 85±8 kJ mol^−1^ for the [SCl_3_]^−^ which is in the same range as the bond energy within the [Cl_3_]^−^ anion (99±5 kJ mol^−1^).^[11]^ In contrast, for the heavier elements Se and Te a plethora of chloroselenates and chlorotellurates in the oxidation states ii and iv are known for decades (e.g., [Se_2_Cl_6_]^2−^,^[12]^ [Se_4_Cl_12_]^2−^,^[13]^ [ChCl_6_]^2−^,^[14]^ [Ch_2_Cl_10_]^2−^,[^13^, ^15^] [Ch_3_Cl_13_]^−^ ^[16]^ (Ch=Se, Te)).

At the outset, we prepared the ionic liquid [NEt_3_Me][Cl_3_] by the reaction of commercially available [NEt_3_Me]Cl with elemental chlorine as previously described.^[3]^

A solution of [NEt_3_Me][Cl_3_] in CH_2_Cl_2_ was reacted with elemental sulfur at room temperature for 16 h [Eq. 1]. Slowly cooling to −40 °C yielded yellow crystals that could be analyzed by X‐ray diffraction revealing the formation of [NEt_3_Me]_2_[SCl_6_] (Figure 1, left).

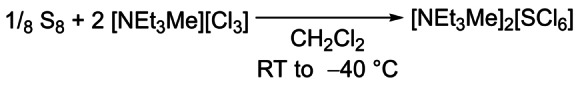




**Figure 1:**
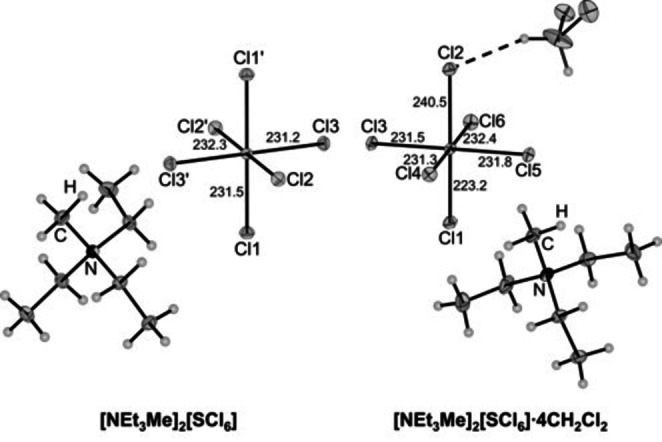
Molecular structure of [NEt_3_Me]_2_[SCl_6_] (left) and [NEt_3_Me]_2_[SCl_6_]⋅4 CH_2_Cl_2_ (right) in the solid state with thermal ellipsoids shown at 50 % probability. Bond lengths are given in pm with an error of 0.1 pm.

In this structure, the [SCl_6_]^2−^ dianion has an almost octahedral geometry with S−Cl bond lengths of 231.2(1), 231.5(1) and 232.3(1) pm and bond angles between 88.9(1)° and 91.1(1)°. The [SCl_6_]^2−^ dianion is stabilized by weak hydrogen bonding interactions between the [NEt_3_Me]^+^ cation and the dianion (see Figure 2). The Raman spectrum of a single crystal of [NEt_3_Me]_2_[SCl_6_] further indicated an octahedral geometry for the [SCl_6_]^2−^ dianion showing only three bands at 282, 241, and 168 cm^−1^ in the region for S−Cl vibrations (See Figure S4). These bands can be assigned to the *A*_1g_ and *E*_g_ symmetric S−Cl stretching vibrations and the *T*_2g_ symmetric bending vibration, respectively, and are in agreement with the vibrational spectra calculated at Cosmo‐SCS‐MP2/def2‐TZVPP level of theory (see Figure S3).

**Figure 2:**
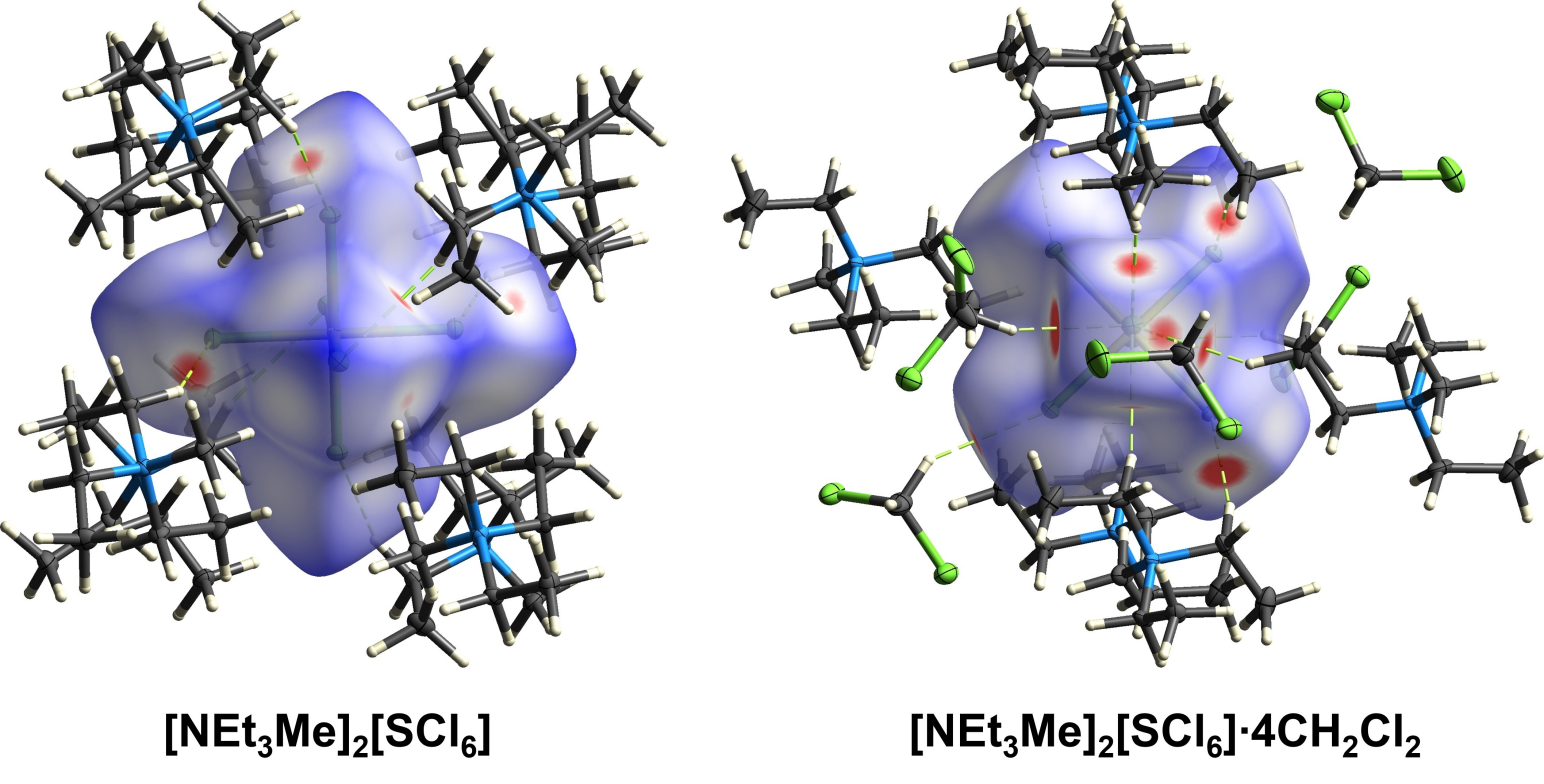
Hirshfeld surface of [NEt_3_Me]_2_[SCl_6_] (left) and [NEt_3_Me]_2_[SCl_6_]⋅4 CH_2_Cl_2_ (right). Disorders are omitted for clarity. Color code: blue=N, grey=C, white=H, yellow=S, green=Cl. Dashed green lines display hydrogen bonds.

When a diluted solution of [NEt_3_Me][Cl_3_] and sulfur in CH_2_Cl_2_ was cooled to −80 °C, co‐crystallization of CH_2_Cl_2_ with [NEt_3_Me]_2_[SCl_6_] was observed. Surprisingly, X‐ray diffraction of the obtained crystals of [NEt_3_Me]_2_[SCl_6_]⋅4 CH_2_Cl_2_ revealed a different structure for the [SCl_6_]^2−^ dianion (Figure 1, right). Compared to the previously analyzed structure, the S−Cl1 bond is shortened by 8.3(1) pm (compared to the average S−Cl bond length) while the S−Cl2 bond is significantly elongated by 8.8(1) pm. Therefore, the octahedral symmetry of the [SCl_6_]^2−^ dianion is distorted yielding a *C*_4v_ symmetric structure.

In general, the [SCl_6_]^2−^ dianion belongs to the group of AB_6_E systems bearing six ligands and one lone pair. According to the valence shell electron pair repulsion model (VSEPR), these systems should possess a stereochemically active lone pair and form structures of distorted octahedral geometries. However, the prediction of the VSEPR model is not always correct for these systems. Consequently, they have received significant attention over the last decades both from an experimental and a theoretical point of view.[^17^, ^18^, ^19^] For XeF_6,_ ^[20]^ [IF_6_]^−^,^[21]^ and [SeF_6_]^2−^ ^[22]^ a distorted octahedral structure with *C*_3v_ symmetry is observed, while, for instance, [ClF_6_]^−^ ^[23]^ and [BrF_6_]^−^ [^22^, ^24^] have octahedral structures. For the higher homologues of [SCl_6_]^2−^, [SeCl_6_]^2−^, ^[25]^ and [TeCl_6_]^2−^ ^[26]^ both regular and distorted octahedral structures were found depending on the corresponding cation.

These examples demonstrate that both structures can be formed by AB_6_E systems. In the literature, two contrary effects are discussed that have to be taken into account. The first effect is that the system can be stabilized in principle when the HOMO and the LUMO are interacting. However, this combination is prohibited in *O*_h_ symmetry due to different irreducible representations of the HOMO (*A*_1g_) and the LUMO (*T*_1u_). When the octahedral structure is distorted, HOMO–LUMO interaction is allowed resulting in a stabilization of the system, also known as a second‐order Jahn–Teller effect. For an octahedral structure the lone pair is located in the s orbital of the central atom and therefore stereochemically inactive. Lowering the symmetry increases the p character of the lone pair. It becomes stereochemically active and can be located at the plane (*C*_3*v*_), edge (*C*_2*v*_), or corner (*C*_4*v*_) of the octahedron (see Figure S8), with the former being the generally favored and the latter the least probable possibility. The second, contrary, effect is the electronic repulsion between the ligands making an octahedral structure more favorable. For many systems, there is a delicate balance between the symmetrical octahedral and the distorted structure. Therefore, already weak interactions of the anion with its molecular environment, e.g., by hydrogen bonding to the cation can determine the structure.[^17^, ^18^]

As mentioned above, the distortion from an *O*_h_ symmetry to a *C*_4*v*_ geometry, as found for [NEt_3_Me]_2_[SCl_6_]⋅4 CH_2_Cl_2_, is quite unusual. Therefore, an NBO analysis of the distorted [SCl_6_]^2−^ was performed and revealed that the lone pair of the sulfur is located in the 3s orbital and is stereochemically inactive (see Figure S9).

To further investigate the energetical influence of a distortion of [SCl_6_]^2−^, a relaxed surface scan was performed by starting from an octahedral structure and subsequently increasing one S−Cl bond (see Figure S10 to S12). An elongation of the S−Cl2 bond by 10 pm translates to an energetical increase of only 0.6 kJ mol^−1^. On the other hand, this distortion leads to an increased negative charge on Cl2 (Natural Charge −0.43 vs. −0.49) which enables stronger hydrogen bonding interactions.

Indeed, these interactions are found by Hirshfeld analysis of the solid state structure of [NEt_3_Me]_2_[SCl_6_]⋅4 CH_2_Cl_2_ (Figure 2). Overall, the Cl2 has five H⋅⋅⋅Cl interactions below the sum of the van der Waals radii (Σ_vdW_(H−Cl)=285 pm).

As one of the S−Cl bonds is significantly elongated, the [SCl_6_]^2−^ species could be described as a [SCl_5_]^−^ fragment and a Cl^−^ anion. In this description, the [SCl_5_]^−^ species consists of four equatorial S−Cl bonds formed by 3‐center‐4‐electron bonds and one axial S−Cl bond which is a classical 2‐center‐2‐electron bond (Figure 3A). Therefore, we calculated the electrostatic potential of [SCl_5_]^−^ and mapped it onto the electron density (Figure 3B). According to these calculations, there is a high negative charge located on the equatorial Cl atoms while a more positive electrostatic potential can be found on the central sulfur atom along the S−Cl_ax_ bond, the so called σ‐hole. Thus, the Cl^−^ can interact with this positively charged region of the [SCl_5_]^−^ fragment. Additionally, the Cl^−^ can donate electron density into the σ* orbital (S−Cl_ax_, LUMO) resulting in a weakening and elongation of the S−Cl_a*x*_ bond. This bonding situation is similar to that found in polyhalides.^[27]^

**Figure 3:**
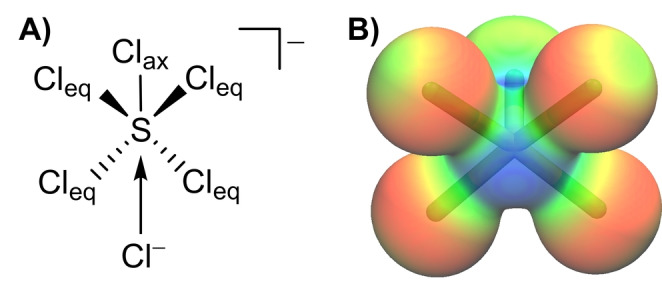
A) Interaction of Cl^−^ with the σ‐hole of [SCl_5_]^−^ formally forming [SCl_6_]^2−^. B) Electrostatic potential of [SCl_5_]^−^ in a range of: −0.15 (red) to 0.05 a.u. (blue) mapped onto the electron density (isosurface value 0.025 a.u.) calculated on B3LYP‐D4/def2‐TZVPP level of theory.

An atoms in molecules (AIM) analysis based on periodic DFT calculations shows an ionic character of the S−Cl interaction in the dianion. At all corresponding bond critical points, the Laplacian is small and positive (ca. 1.7–1.8 e Å^−5^), the ratio of the absolute potential (|V|) and kinetic energy density (G) is below 2.0 (1.6), and the value of the electron localization function (ELF) is only about 0.6—all indicative for a non‐shared interaction (see Table S5).

At last, we investigated the influence of the temperature on the formation of [NEt_3_Me]_2_[SCl_6_] from a stoichiometric mixture of S_8_ and [NEt_3_Me][Cl_3_] by Raman spectroscopy (Figure 4). Interestingly, at 40 °C the mixture exists as a liquid consisting of various species including [Cl_3_]^−^ and S_2_Cl_2_ as indicated by comparison with reference substances (see Figure S6). In addition, the presence of [SCl_3_]^−^ could be assumed as the calculated spectrum at SCS‐MP2/def2‐TZVPP level of theory is in good agreement with the measured spectrum (see Figure S6). When cooled to room temperature, the mixture solidified and the measured Raman spectrum thereof was consistent with those obtained for the single crystals of [NEt_3_Me]_2_[SCl_6_] suggesting its selective formation. This highlights that the crystallization of [NEt_3_Me]_2_[SCl_6_] is energetically highly favored due to its comparably large lattice energy. A rough estimation of the stabilization energy the dianion meets due to the periodic cation‐lattice yields about 240 kJ mol^−1^ (see Supporting Information, chapter h). When the solidified [NEt_3_Me]_2_[SCl_6_] is heated to 40 °C again, a similar spectrum is observed as for the reaction mixture. Thus, it can be assumed that there is an equilibrium between various sulfur chlorides in the liquid mixture and [SCl_6_]^2−^ in the solid (Figure 4A). Given these results, the thermal behavior of [NEt_3_Me]_2_[SCl_6_] is comparable to SCl_4_, which is only stable below −30 °C and decomposes above this temperature to SCl_2_ and Cl_2_.

**Figure 4:**
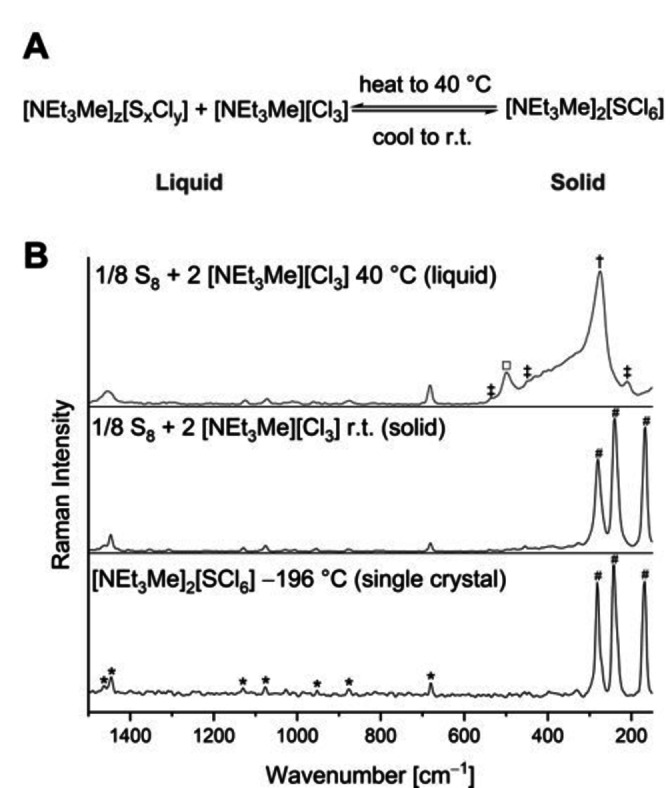
A) The thermal equilibrium of [NEt_3_Me]_2_[SCl_6_] with various sulfur chlorine compounds. B) Raman spectrum of the reaction mixture of sulfur and [NEt_3_Me][Cl_3_] at 40 °C (above) and room temperature (middle) and comparison to the Raman spectrum of the single crystal of [NEt_3_Me]_2_[SCl_6_] (below). Bands are assigned as follows (*=cation, #=[SCl_6_]^2−^, †=[Cl_3_]^−^ ≠=S_2_Cl_2_, □=presumably [SCl_3_]^−^). See Figure S6 for reference spectra.

In conclusion, we synthesized [NEt_3_Me]_2_[SCl_6_], which is the first example of a halosulfate anion of the type [S_*x*_X_*y*_]^*z*−^. Additionally, this unprecedented molecule is one of the few examples for a 14‐valence electron AB_6_E system with a central atom of the third period. In general, AB_6_E systems can either adopt an octahedral symmetry or a distorted structure with a stereochemically active lone pair. In [NEt_3_Me]_2_[SCl_6_], we found an octahedral symmetry for the [SCl_6_]^2−^ dianion in the solid‐state structure. However, when CH_2_Cl_2_ co‐crystallized, [NEt_3_Me]_2_[SCl_6_]⋅4 CH_2_Cl_2_ was formed with an unusual *c*_4*v*_ structure, with one elongated and one shortened axial S−Cl bond. Quantum‐chemical calculations revealed that this distortion cannot be attributed to a stereochemically active lone pair but is a result of enhanced hydrogen bonding interactions with CH_2_Cl_2_.

Notably, [NEt_3_Me]_2_[SCl_6_] decomposes to various sulfur chlorine compounds at 40 °C while cooling back to room temperature results in the selective formation of [NEt_3_Me]_2_[SCl_6_] highlighting the similarity to the well‐studied SCl_4_. This work demonstrates the unique ability of [NEt_3_Me][Cl_3_] to serve as a versatile chlorination agent while stabilizing unprecedent anions.

## Conflict of interest

The authors declare no conflict of interest.

## Supporting information

As a service to our authors and readers, this journal provides supporting information supplied by the authors. Such materials are peer reviewed and may be re‐organized for online delivery, but are not copy‐edited or typeset. Technical support issues arising from supporting information (other than missing files) should be addressed to the authors.

Supporting Information

Supporting Information

Supporting Information

## Data Availability

The data that support the findings of this study are available from the corresponding author upon reasonable request.

## References

[1a] R. Lin , A. P. Amrute , J. Pérez-Ramírez , Chem. Rev. 2017, 117, 4182; 28150944 10.1021/acs.chemrev.6b00551

[1b] P. Schmittinger , T. Florkiewicz , L. C. Curlin , B. Lüke , R. Scannell , T. Navin , E. Zelfel , R. Bartsch , in Ullmann's Encyclopedia of Industrial Chemistry (Chlorine), Wiley-VCH, Weinheim, 2011.

[2] P. Voßnacker , N. Schwarze , T. Keilhack , M. Kleoff , S. Steinhauer , Y. Schiesser , M. Paven , S. Yogendra , R. Weber , S. Riedel , ACS Sustainable Chem. Eng. 2022, 10, 9525.

[3] P. Voßnacker , A. Wüst , T. Keilhack , C. Müller , S. Steinhauer , H. Beckers , S. Yogendra , Y. Schiesser , R. Weber , M. Reimann , et al., Sci. Adv. 2021, 7, eabj5186. 34586844 10.1126/sciadv.abj5186 PMC8480918

[4a] P. Pröhm , J. R. Schmid , K. Sonnenberg , S. Steinhauer , C. J. Schattenberg , R. Müller , M. Kaupp , P. Voßnacker , S. Riedel , Angew. Chem. Int. Ed. 2020, 59, 16002; 10.1002/anie.202006268 PMC7540313 32459871

[4b] J. R. Schmid , P. Pröhm , P. Voßnacker , G. Thiele , M. Ellwanger , S. Steinhauer , S. Riedel , Eur. J. Inorg. Chem. 2020, 4497;

[4c] T. A. Gully , P. Voßnacker , J. R. Schmid , H. Beckers , S. Riedel , ChemistryOpen 2021, 10, 255; 33507623 10.1002/open.202000263 PMC7874256

[4d] S. Kotsyuda , A. Wiesner , S. Steinhauer , S. Riedel , Z. Anorg. Allg. Chem. 2020, 57, 13982.

[5] M. Baudler , G. Brauer , Handbuch der präparativen anorganischen Chemie in drei Bänden, Vol. 1 , 3rd ed., Ferdinand Enke, Stuttgart, 1975.

[6a] R. Steudel , D. Jensen , B. Plinke , Z. Naturforsch. B 1987, 42, 163;

[6b] R. Kniep , L. Körte , D. Mootz , Z. Naturforsch. B 1984, 39, 305.

[7a] A. J. Edwards , J. Chem. Soc. Dalton Trans. 1978, 1723;

[7b] A. Finch , P. N. Gates , T. H. Page , Inorg. Chim. Acta 1977, 25, L49–L50.

[8] J. Passmore , P. D. Boyle , G. Schatte , T. Way , T. S. Cameron , Can. J. Chem. 1996, 74, 1671.

[9] P. Weis , D. C. Röhner , R. Prediger , B. Butschke , H. Scherer , S. Weber , I. Krossing , Chem. Sci. 2019, 10, 10779. 32055385 10.1039/c9sc03915e PMC7006506

[10] D. Dirican , N. Pfister , M. Wozniak , T. Braun , Chem. Eur. J. 2020, 26, 6945. 31840851 10.1002/chem.201904493 PMC7318666

[11a] B. D. Gailbreath , C. A. Pommerening , S. M. Bachrach , L. S. Sunderlin , J. Phys. Chem. A 2000, 104, 2958;

[11b] K. E. Nizzi , C. A. Pommerening , L. S. Sunderlin , J. Phys. Chem. A 1998, 102, 7674.

[12] B. Krebs , E. Lührs , R. Willmer , F.-P. Ahlers , Z. Anorg. Allg. Chem. 1991, 592, 17.

[13] W. Czado , M. Maurer , U. Müller , Z. Anorg. Allg. Chem. 1998, 624, 1871.

[14] P. J. Hendra , Z. Jovi , J. Chem. Soc. A 1968, 600.

[15] M. A. James , O. Knop , T. S. Cameron , Can. J. Chem. 1992, 70, 1795.

[16] F.-P. Ahlers , E. Lührs , B. Krebs , Z. Anorg. Allg. Chem. 1991, 594, 7.

[17] R. A. Wheeler , P. N. V. P. Kumar , J. Am. Chem. Soc. 1992, 114, 4776.

[18] M. Kaupp , C. van Wuellen , R. Franke , F. Schmitz , W. Kutzelnigg , J. Am. Chem. Soc. 1996, 118, 11939.

[19] M. Gawrilow , H. Beckers , S. Riedel , L. Cheng , J. Phys. Chem. A 2018, 122, 119. 29220184 10.1021/acs.jpca.7b09902

[20] J. N. Cutler , G. M. Bancroft , J. D. Bozek , K. H. Tan , G. J. Schrobilgen , J. Am. Chem. Soc. 1991, 113, 9125.

[21] A. R. Mahjoub , K. Seppelt , Angew. Chem. 1991, 103, 309.

[22] A. R. Mahjoub , X. Zhang , K. Seppelt , Chem. Eur. J. 1995, 1, 261.

[23] K. O. Christe , W. W. Wilson , R. V. Chirakal , J. C. P. Sanders , G. J. Schrobilgen , Inorg. Chem. 1990, 29, 3506.

[24] A. R. Mahjoub , A. Hoser , J. Fuchs , K. Seppelt , Angew. Chem. Int. Ed. Engl. 1989, 28, 1526;

[25a] W. Abriel , Acta Crystallogr. Sect. C 1986, 42, 1113;

[25b] U. Müller , B. Eckhoff , Z. Kristallogr. New Cryst. Struct. 1999, 214, 505.

[26] W. Abriel , Z. Naturforsch. B 1986, 41, 592.

[27] K. Sonnenberg , L. Mann , F. A. Redeker , B. Schmidt , S. Riedel , Angew. Chem. Int. Ed. 2020, 59, 5464–5493; 10.1002/anie.201903197 31090163

