# Supplementary material for: Synthesis of a Hexachloro Sulfate(IV) Dianion Enabled by Polychloride Chemistry
Source: Angew Chem Int Ed Engl. 2022 Aug 30;61(43):e202209684. doi: 10.1002/anie.202209684 (PMC9805220; doi:10.1002/anie.202209684)
Supplement: Supplementary file 3 — Supporting Information [file ANIE-61-0-s002.pdf]

Angewandte Chemie
Eine Zeitschrift der Gesellschaft Deutscher Chemiker

Supporting Information

Synthesis of a Hexachloro Sulfate(IV) Dianion Enabled by Polychloride Chemistry

*P. Voßnacker, A. Wüst, C. Müller, M. Kleoff, S. Riedel**

Table of Contents

a) Experimental Section 2

a1. Apparatus and Materials 2

a2. Synthesis of $[NEt_3Me]_2[SCl_6]$ 2

a3. Synthesis of $[NEt_3Me]_2[SCl_6]\cdot 4CH_2Cl_2$ 3

a4. Stability of $[NEt_3Me]_2[SCl_6]$ 3

b) Molecular Structures in Solid State Including Intermolecular Interactions 4

b1. $[NEt_3Me]_2[SCl_6]$ 4

b2. $[NEt_3Me]_2[SCl_6]\cdot 4\ CH_2Cl_2$ 5

c) Crystal Data 6

d) Experimental and Calculated Raman and IR Spectra 7

e) Stereochemically Active Lone Pair in AB_6E Systems 10

f) NBO Analysis of Symmetric and Asymmetric $[SCl_6]^{2-}$ 11

g) Relaxed Surface Scan of $[SCl_6]^{2-}$ 13

h) Solid State Calculations 16

i) Optimized Structures 19

j) Calculated Energies and Free Reaction Energies 21

j1) B3LYP(D4)/def2-TZVPP Energies 21

j2) SCS-MP2/def2-TZVPP Energies 22

j3) Free Reaction Energy Calculation 23

k) Calculated Vibrational Frequencies 25

l) Coordinates of Optimized Structures 34

m) References 44

a) Experimental Section

a1. Apparatus and Materials

All substances sensitive to water and oxygen were handled under an argon atmosphere using standard Schlenk techniques and oil pump vacuum up to 10^{-3} mbar. Commercially available $[NEt_3Me]Cl$, sulfur, and chlorine were used without further purification. All salts were dried *in vacuo* at 100 °C for 1 h to 1 day prior to use. Dry DCM was obtained by storage over activated 3 Å molecular sieves. Raman spectra were recorded at room temperature on a Bruker (Karlsruhe, Germany) MultiRAM II equipped with a low-temperature Ge detector (1064 nm, 100-180 mW, resolution of 4 cm^{-1}). Spectra of single crystals were recorded at –196 °C using the Bruker RamanScope III (See *Chem.Eur.J.* **2020**,*26*,13256–13263 for detailed description) [1] X-ray diffraction data were collected on a Bruker D8 Venture CMOS area detector (Photon 100) diffractometer with $Mo_{K\alpha}$ radiation. Single crystals were coated with perfluoroether oil at low temperature (–40/–80 °C) and mounted on a 0.1-0.2 mm Micromount. The structures were solved with the ShelXT[2] structure solution program using intrinsic phasing and refined with the ShelXL[3] refinement package using least squares on weighted F2 values for all reflections using OLEX2[4]. Hydrogen atom were treated using the HFIX 23 (CH_2 groups) and HFIX 137 (for CH_3 groups) restrains as implemented in ShelXL. For structure optimization (with and without solvent model COSMO[5]), and frequency calculations (including Raman intensities) the program package TURBOMOLE 7.3[6] was used. Relaxed surface scan were performed using orca 5.0.3[7] and NBO[8] analyses were performed with Gaussain G16[9] software package. Functionals (B3LYP(D4)[10,11] and SCS-MP2[12]) and the basis set (def2-TZVPP[13], aug-cc-pVTZ[14]) were used as implemented. Minima on the potential energy surface were characterized by harmonic vibrational frequency analysis. Thermochemistry was provided for ΔG values calculated at 298.15 K and 1.0 bar. Representations of Hirshfeld surfaces were generated using CrystalExplorer21[15]

a2. Synthesis of $[NEt_3Me]_2[SCl_6]$

270 mg (1.06 mmol, 1.35 equiv. (1.54 mmol Cl_2, 1.97 equiv.) $[NEt_3Me][Cl(Cl_2)_{1.45}]$ was dissolved in 1.5 mL CH_2Cl_2 and 25 mg (0.78 mmol, 1 equiv.) sulfur was added. A clear solution was obtained which was stirred overnight. Yellow single crystals of $[NEt_3Me]_2[SCl_6]$ were obtained within several days by slowly cooling to –40 °C.

$[NEt_3Me]_2[SCl_6]$ Raman (–196 °C): $\tilde{\nu}$ = 3023 (w), 2994 (m), 2941 (m), 1446 (w), 680 (w), 3016 (w), 2973 (w), 1076 (w), 877 (vw), 281 (vs), 242 (vs), 168 (vs) cm^{-1}.

CCDC number: 2156516

a3. Synthesis of $[NEt_3Me]_2[SCl_6]\cdot 4CH_2Cl_2$

75 mg (1.06 mmol, 2 equiv.) Cl_2 was condensed onto 160 mg (1.06 mmol, 2 equiv. $[NEt_3Me]Cl$ and 16 mg (0.53 mmol, 1 equiv.) sulfur. Addition of 2 mL CH_2Cl_2 yields a clear solution. Yellow single crystals of $[NEt_3Me]_2[SCl_6]\cdot 4CH_2Cl_2$ were obtained by slowly cooling to –80 °C.

$[NEt_3Me][SCl_6]$ Raman (–196 °C): ṽ = 3043 (w), 2987 (m), 2976 (m), 2958 (w), 2936 (w), 1474 (w), 1144 (w), 699 (m), 345 (w), 277 (vs), 239 (vs), 177 (m), 166 (w) cm^{-1}.

CCDC number: 2156517

a4. Stability of $[NEt_3Me]_2[SCl_6]$

0.466 g (6.57 mmol, 1.93 equiv.) Cl_2 was condensed onto 1.035 g (6.82 mmol, 2 equiv.) $[NEt_3Me]Cl$ and 0.109 g (3.40 mmol, 1 equiv.) sulfur. The reaction mixture was warmed to 40 °C and a clear orange solution was obtained. Cooling to room temperature yields an orange solid. The reaction mixture was characterized by Raman spectroscopy at room temperature and 40 °C and by IR spectroscopy at –80 °C.

Raman (RT): ṽ = 3016 (w), 2991 (m), 2945 (m), 2885 (vw), 1448 (w), 1129 (vw), 1075 (vw), 1027 (vw), 1009 (vw), 956 (vw), 877 (vw), 680 (w), 451 (vw), 278 (vs), 237 (vs), 166 (vs) cm^{-1}.

Raman (40 °C): ṽ = 2985 (m), 2946 (m), 1455 (vw), 1124 (vw), 1072 (vw), 1011 (vw), 961 (vw), 875 (vw), 682 (w), 499 (m), 534 (vw), 429 (m), 275 (vs), 210 (w) cm^{-1}.

IR (–80 °C): ṽ = 3024 (vw), 2983 (w), 2946 (vw), 1486 (w), 1444 (m), 1402 (m), 1393 (w), 1379 (w), 1379 (w), 1370 (w), 1350 (w), 1317 (w), 1305 (vw), 1213 (w), 1205 (w), 1205 (w), 1191 (w), 1151 (vw), 1127 (w), 1071 (vw), 1024 (m), 1007 (w), 954 (w), 873 (vw), 808 (w), 791 (m), 305 (vs), 264 (s), 126 (vs) cm^{-1}.

b) Molecular Structures in Solid State Including Intermolecular Interactions

b1. $[NEt_3Me]_2[SCl_6]$

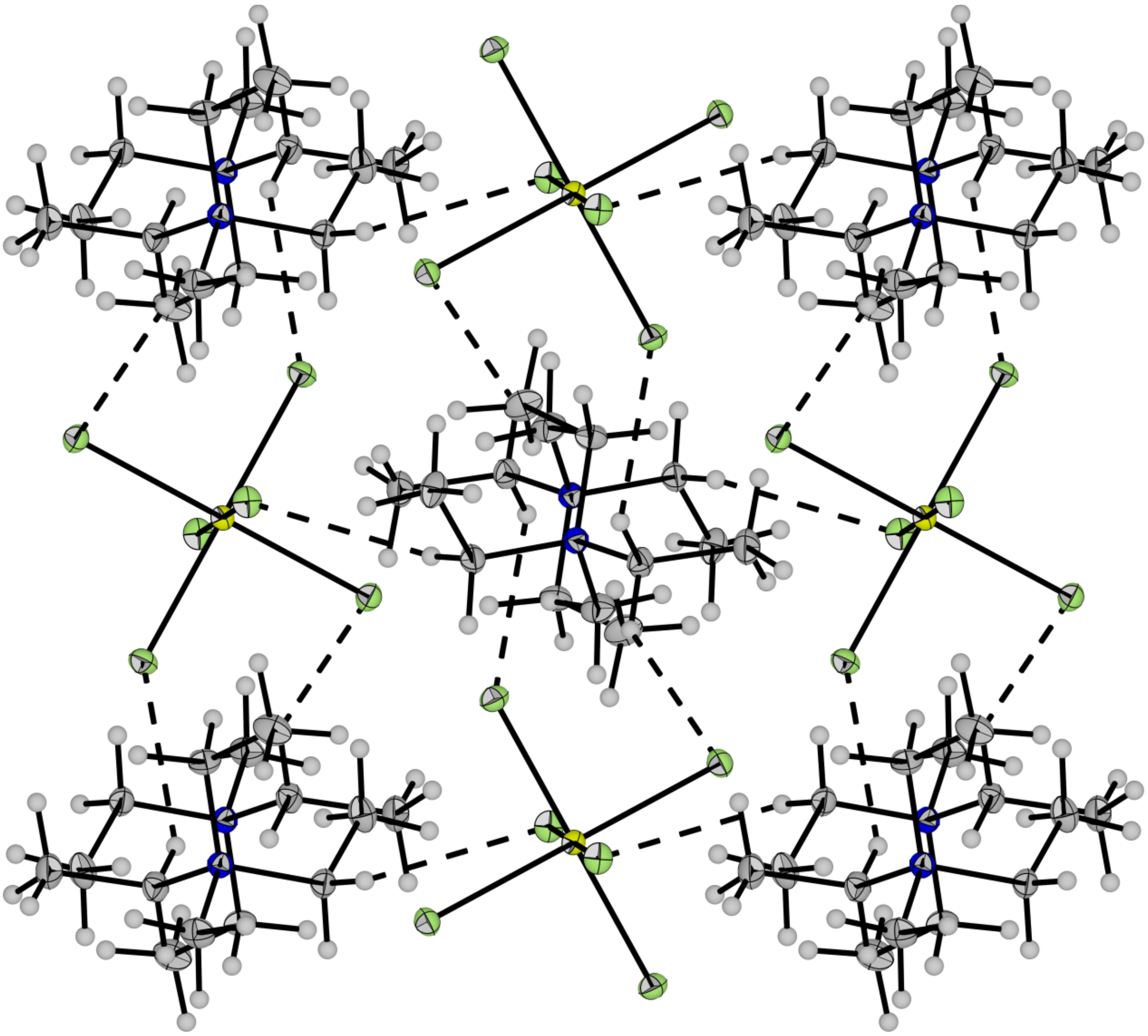

Figure S 1. Arrangement of the molecules within the unit cell of $[NEt_3Me]_2[SCl_6]$. Thermal ellipsoids are shown at 50 % probability. Cl-H hydrogen bonds are displayed by dashed lines.

b2. $[NEt_3Me]_2[SCl_6]\cdot 4\ CH_2Cl_2$

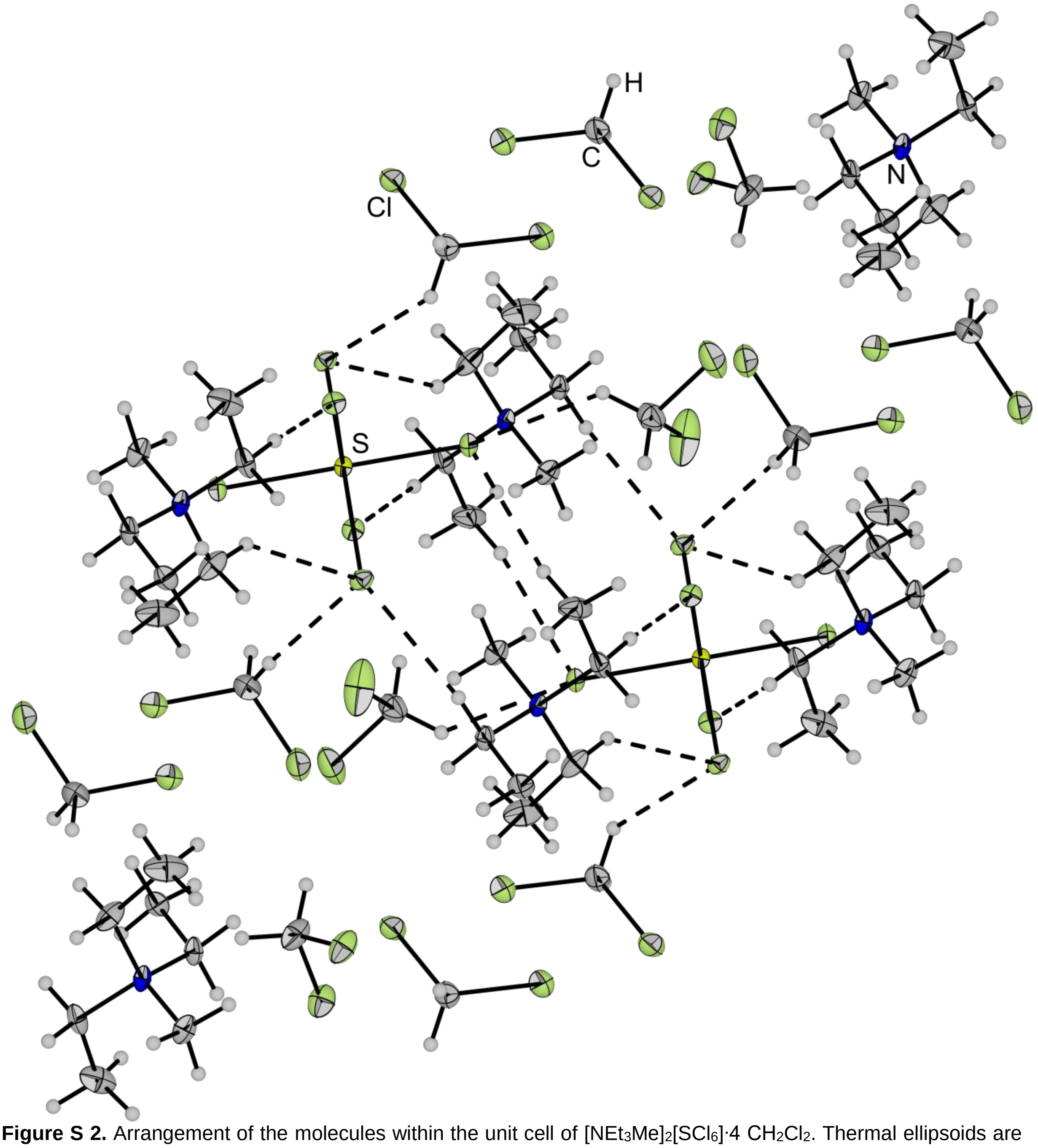

Figure S 2. Arrangement of the molecules within the unit cell of $[NEt_3Me]_2[SCl_6]\cdot 4\ CH_2Cl_2$. Thermal ellipsoids are shown at 50 % probability. Cl-H hydrogen bonds are displayed by dashed lines. Disorder is omitted for clarity.

c) Crystal Data

Table S 1. Crystal data of the synthesized compounds.

Empirical formula	$C_{14}H_{36}Cl_6N_2S$	$C_{18}H_{44}Cl_{14}N_2S$
Formula weight	477.21	816.91
Temperature/K	104.4	100.0
Crystal system	monoclinic	triclinic
Space group	$P2_1/n$	P-1
a/Å	9.7726(4)	10.1220(10)
b/Å	9.0349(5)	13.5938(9)
c/Å	13.1501(6)	14.6209(15)
α/°	90	116.305(3)
β/°	90.136(2)	90.917(3)
γ/°	90	91.176(3)
Volume/$Å^3$	1161.08(10)	1802.4(3)
Z	2	2
$\rho_{calc}g/cm^3$	1.365	1.505
μ/mm^{-1}	0.831	1.143
F(000)	504.0	840.0
Crystal size/mm^3	0.387 × 0.312 × 0.242	0.502 × 0.452 × 0.335
Radiation	MoKα (λ = 0.71073)	MoKα (λ = 0.71073)
2Θ range for data collection/°	5.2 to 61.024	4.026 to 56.608
Reflections collected	47821	122213
Independent reflections	3496 [R_{int} = 0.0291, R_{sigma} = 0.0125]	8950 [R_{int} = 0.0602, R_{sigma} = 0.0264]
Data/restraints/parameters	3496/0/110	8950/12/373
Goodness-of-fit on F^2	1.129	1.024
Final R indexes [I>=2σ (I)]	R_1 = 0.0173, wR_2 = 0.0425	R_1 = 0.0252, wR_2 = 0.0579
Final R indexes [all data]	R_1 = 0.0202, wR_2 = 0.0443	R_1 = 0.0336, wR_2 = 0.0610
Largest diff. peak/hole / e $Å^{-3}$	0.40/-0.22	0.38/-0.29
CCDC deposition numbers	2156516	2156517

d) Experimental and Calculated Raman and IR Spectra

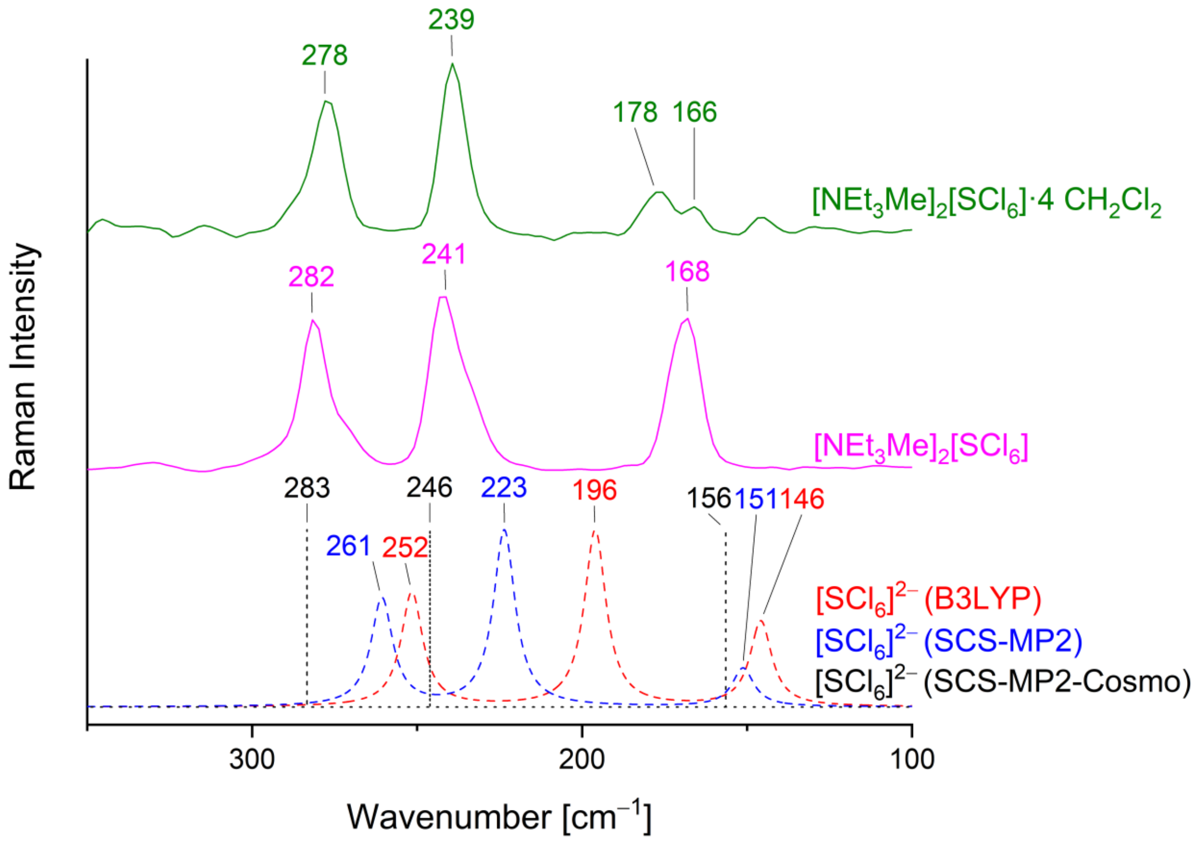

Figure S 3. Experimental Raman spectrum of a single crystal of $[NEt_3Me]_2[SCl_6]$ and $[NEt_3Me]_2[SCl_6]\cdot 4\ CH_2Cl_2$ recorded at –196 °C and comparison to the calculated spectra (B3LYP/def2-TZVPP, SCS-MP2/def2-TZVPP and SCS-MP2-Cosmo/def2-TZVPP (ε_r = 100) level of theory).

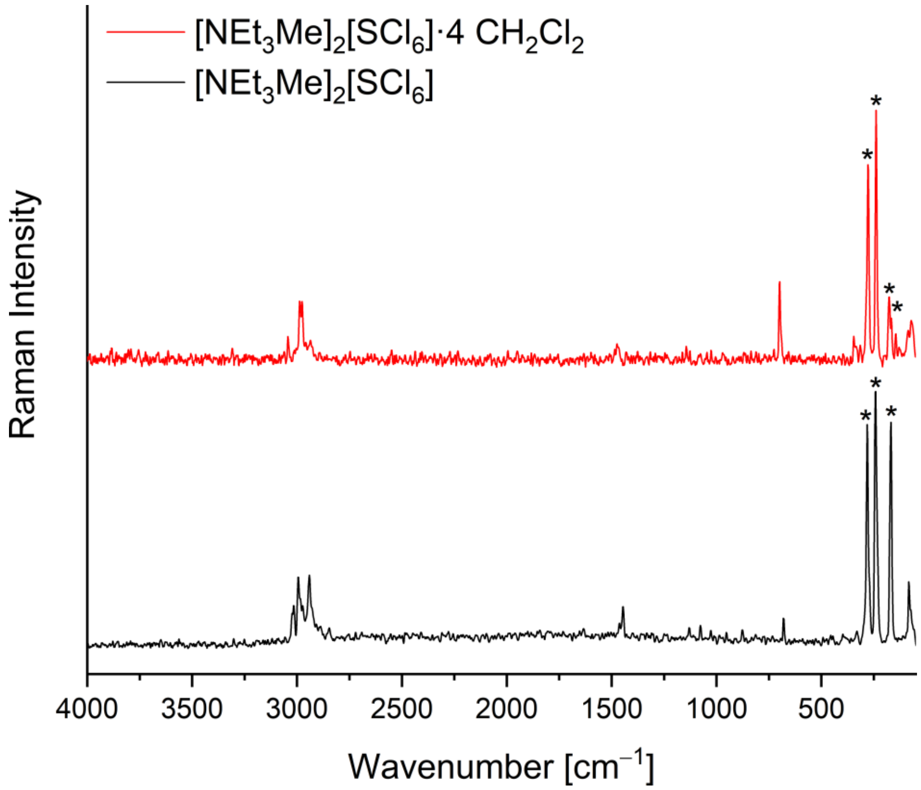

Figure S 4. Experimental Raman spectrum of a single crystal of $[NEt_3Me]_2[SCl_6]$ and $[NEt_3Me]_2[SCl_6]{\cdot}4\ CH_2Cl_2$ recorded at –196 °C. Bands highlighted with asterisk are associated to the $[SCl_6]^{2-}$.

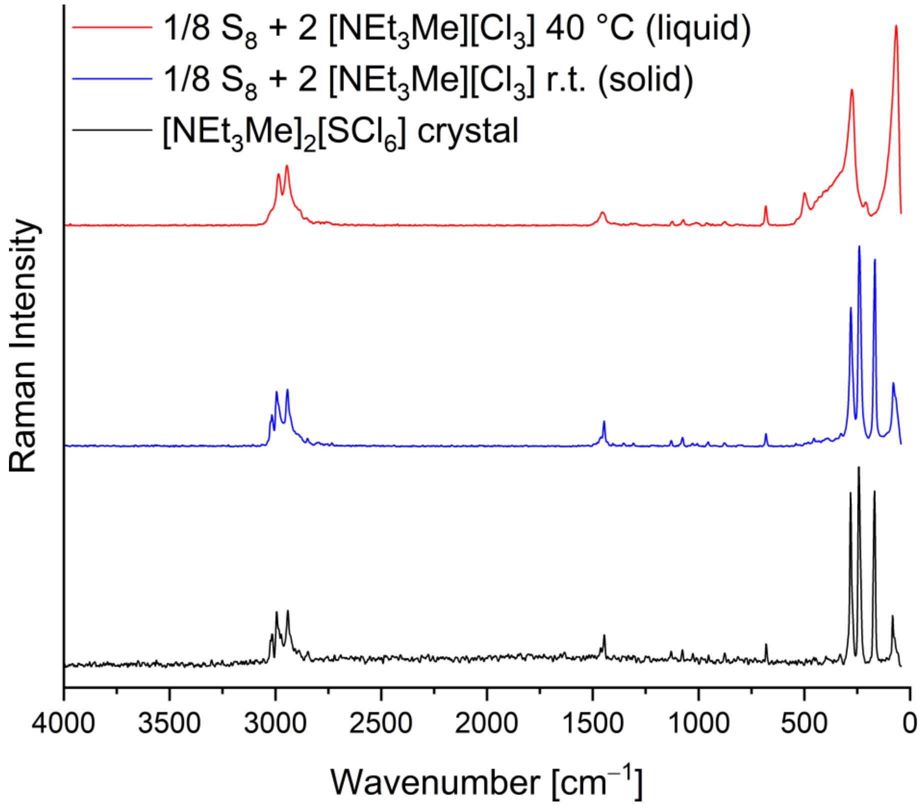

Figure S 5. Raman spectrum of the reaction mixture of sulfur and $[NEt_3Me][Cl_3]$ at 40 °C (red) and room temperature (blue) and comparison to the Raman spectrum of the single crystal of $[NEt_3Me]_2[SCl_6]$ (black).

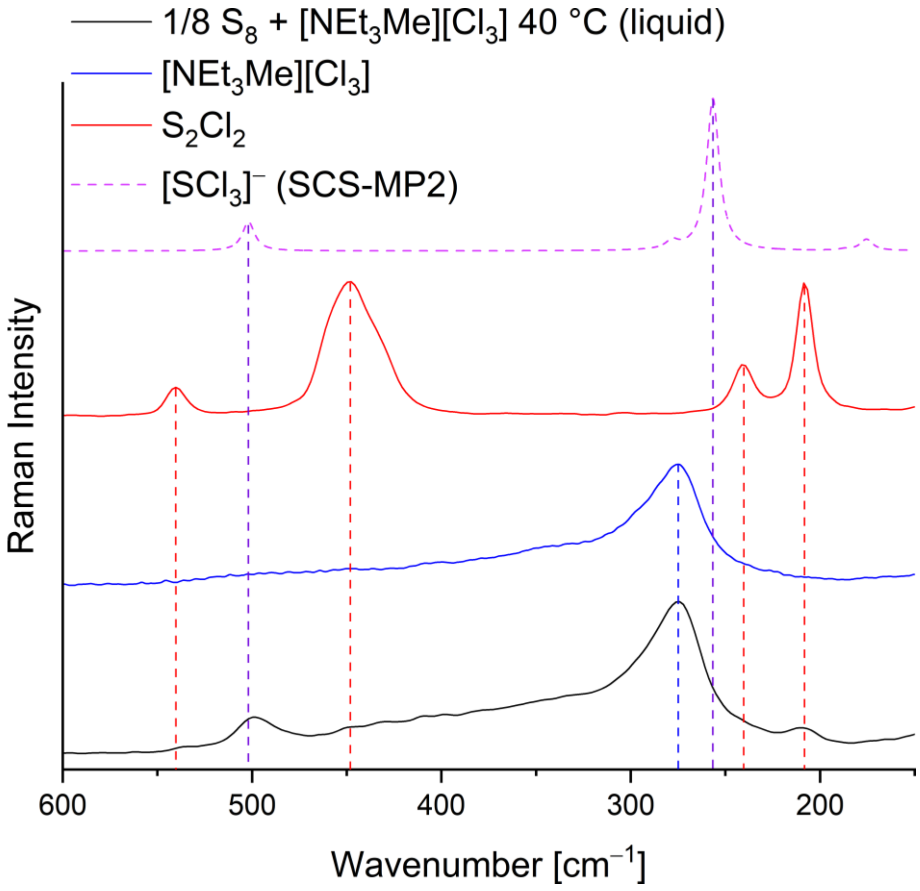

Figure S 6. Raman spectra of the reaction mixture of sulfur and $[NEt_3Me][Cl_3]$ at 40 °C as well as reference spectra of $[NEt_3Me][Cl_3]$, S_2Cl_2 and the calculated spectrum of $[SCl_3]^-$ (SCS-MP2/def2-TZVPP).

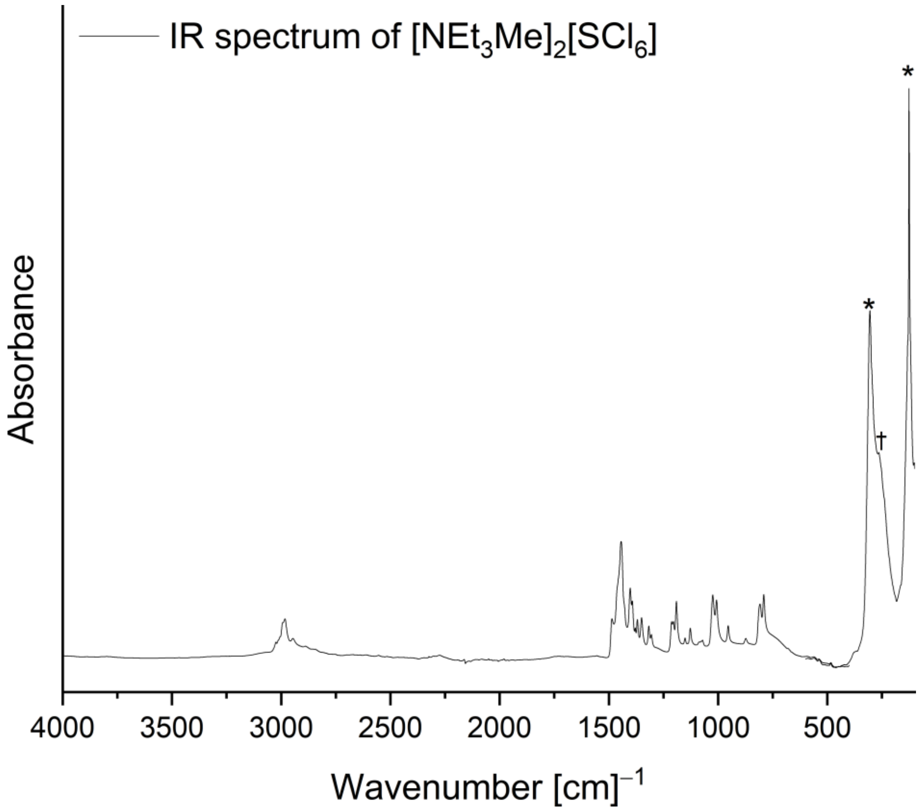

Figure S 7. IR spectrum of the reaction mixture of sulfur and $[NEt_3Me][Cl_3]$ recorded at –80 °C. Bands highlighted with an asterisk belong to the $[SCl_6]^{2-}$ anion while the band highlighted with a dagger presumably corresponds to $[SCl_3]^-$.

e) Stereochemically Active Lone Pair in AB_6E Systems

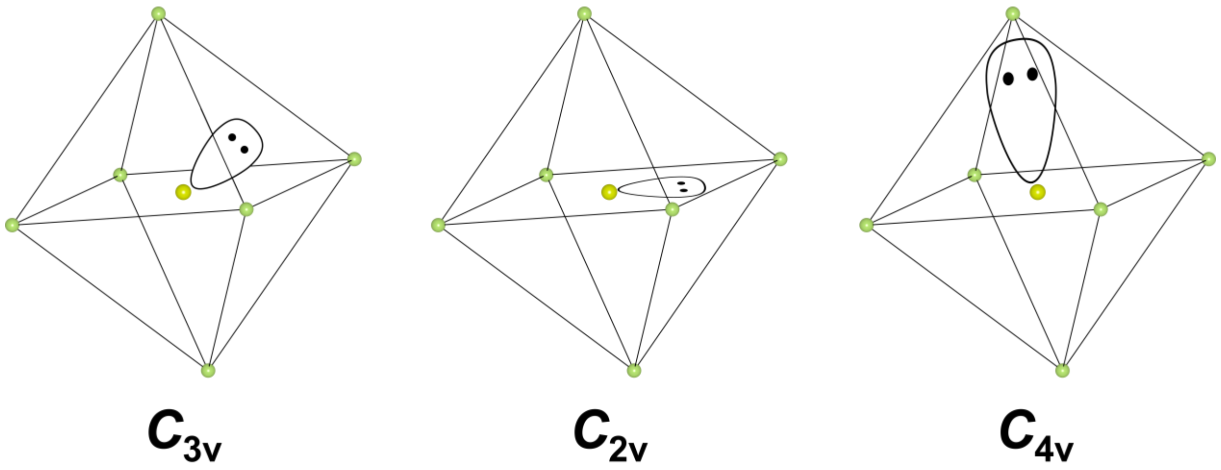

Figure S 8. Possible orientations of the stereochemically active lone pair in AB_6E systems.

f) NBO Analysis of Symmetric and Asymmetric $[SCl_6]^{2-}$

NBO analysis for $[SCl_6]^{2-}$ was performed on B3LYP/aug-cc-pVTZ level of theory using the NBO 7.0.4 program as implemented in Gaussian 16. The NBO analysis was performed on geometries of $[SCl_6]^{2-}$ taken from the solid state structures of $[NEt_3Me]_2[SCl_6]$ and $[NEt_3Me]_2[SCl_6]\cdot 4CH_2Cl_2$. For the symmetric $[SCl_6]^{2-}$ ($[NEt_3Me]_2[SCl_6]$) the lone pair has a s-character of 100% while for the asymmetric $[SCl_6]^{2-}$ ($[NEt_3Me]_2[SCl_6]\cdot 4CH_2Cl_2$) the lone pair has a s-character of 99.67 % and a p-character of 0.33 %.

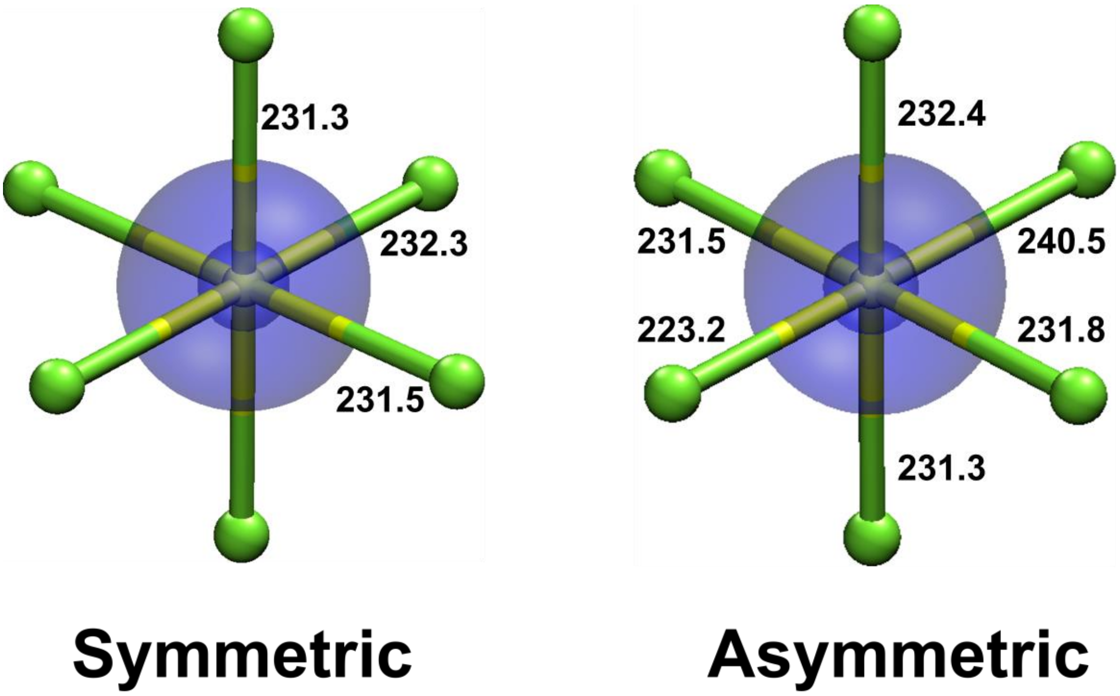

Figure S 9. NBOs showing the lone pair located at the sulfur atom for the symmetric $[SCl_6]^{2-}$ ($[NEt_3Me]_2[SCl_6]$, left) and the asymmetric $[SCl_6]^{2-}$ ($[NEt_3Me]_2[SCl_6]\cdot 4CH_2Cl_2$, right). Isosurface value 0.1 a.u., bond lengths are given in pm.

Table S 2. Summary of Natural Population Analysis for the symmetric $[SCl_6]^{2-}$.

Atom	No	Natural Charge	Natural Population			
			Core	Valence	Rydberg	Total
S	1	0.56732	9.99999	5.29113	0.14156	15.43268
Cl	2	–0.42760	9.99999	7.41535	0.01226	17.42760
Cl	3	–0.42696	9.99999	7.41461	0.01236	17.42696
Cl	4	–0.42910	9.99999	7.41706	0.01204	17.42910
Cl	5	–0.42760	9.99999	7.41535	0.01226	17.42760
Cl	6	–0.42696	9.99999	7.41461	0.01236	17.42696
Cl	7	–0.42910	9.99999	7.41706	0.01204	17.42910
Total	-	–2	69.99996	49.78516	0.21487	120

Table S 3. Summary of Natural Population Analysis for the asymmetric $[SCl_6]^{2-}$.

Atom	No	Natural Charge	Natural Population			
			Core	Valence	Rydberg	Total
Cl	1	–0.42877	9.99999	7.41662	0.01215	17.42877
S	2	0.57218	9.99999	5.2858	0.14202	15.42782
Cl	3	–0.42451	9.99999	7.41213	0.01238	17.42451
Cl	4	–0.36797	9.99999	7.35176	0.01621	17.36797
Cl	5	–0.42673	9.99999	7.41446	0.01228	17.42673
Cl	6	–0.49245	10.00000	7.48344	0.00901	17.49245
Cl	7	–0.43176	9.99999	7.41984	0.01193	17.43176
Total	-	–2	69.99996	49.78405	0.21599	120

Coordinates for the symmetric $[SCl_6]^{2-}$

S –4.88630 9.03490 0.00000

Cl –3.78193 7.03123 –0.35558

Cl –4.54947 8.81282 2.27654

Cl –6.92342 7.93165 0.17161

Cl –5.99067 11.03857 0.35558

Cl –5.22313 9.25698 –2.27654

Cl –2.84918 10.13815 –0.17161

Coordinates for the asymmetric $[SCl_6]^{2-}$

Cl 4.36174 2.94997 3.48244

S 2.35235 1.80829 3.30268

Cl 2.37056 1.41420 5.58199

Cl 3.44747 –0.11057 2.97750

Cl 0.35165 0.65522 3.13551

Cl 1.15929 3.86980 3.63757

Cl 2.32435 2.21746 1.01486

g) Relaxed Surface Scan of $[SCl_6]^{2-}$

The relaxed surface scan for $[SCl_6]^{2-}$ was performed on B3LYP/def2-TZVPP level of theory using a CPCM model for CH_2Cl_2 as implemented in orca 5.0.3. The scan was started at the equilibrium structure of $[SCl_6]^{2-}$ and one S-Cl bond was elongated in 5 pm increments while all other degrees of freedom were optimizing freely.

Table S 4. Calculated Energies for the relaxed surface scan of $[SCl_6]^{2-}$.

R (S-Cl1)	*E* (Hatree)	Δ*E* (kJ mol^{-1})
237.63	-3159.43917236	0.00
242.63	-3159.43910991	0.16
247.63	-3159.43894456	0.60
252.63	-3159.43869710	1.25
257.63	-3159.43838824	2.06
262.63	-3159.43804388	2.96
267.63	-3159.43767931	3.92
272.63	-3159.43729111	4.94
277.63	-3159.43686877	6.05
282.63	-3159.43640691	7.26
287.63	-3159.43591142	8.56
292.63	-3159.43539777	9.91
297.63	-3159.43488392	11.26
302.63	-3159.43438382	12.57
307.63	-3159.43390545	13.83
312.63	-3159.43345178	15.02
317.63	-3159.43302258	16.15
322.63	-3159.43261865	17.21
327.63	-3159.43224617	18.18
332.63	-3159.43191625	19.05
337.63	-3159.43163663	19.79
342.63	-3159.43140226	20.40
347.63	-3159.43119659	20.94
352.63	-3159.43100297	21.45
357.63	-3159.43081470	21.94
362.63	-3159.43063880	22.40
367.63	-3159.43048405	22.81
372.63	-3159.43033711	23.20
377.63	-3159.43018948	23.58
382.63	-3159.43006028	23.92
387.63	-3159.42996910	24.16
392.63	-3159.42990485	24.33
397.63	-3159.42984703	24.48
402.63	-3159.42979206	24.63
407.63	-3159.42974122	24.76
412.63	-3159.42969771	24.88
417.63	-3159.42967816	24.93
422.63	-3159.42970806	24.85
427.63	-3159.42975366	24.73
432.63	-3159.42977422	24.67
437.63	-3159.42978394	24.65

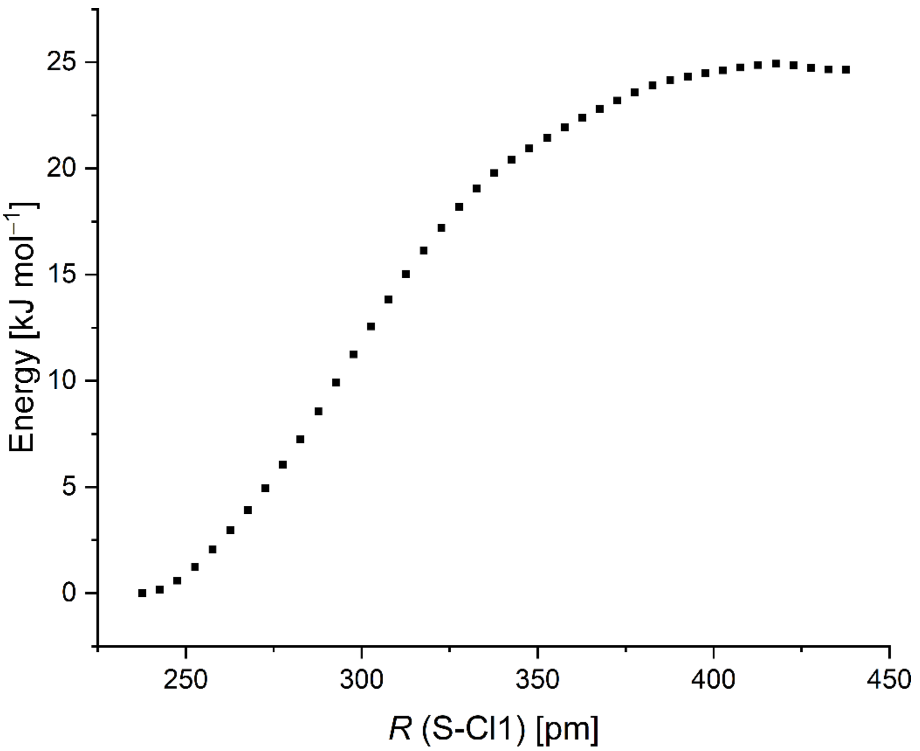

Figure S 10. Relaxed surface scan for $[SCl_6]^{2-}$ (complete).

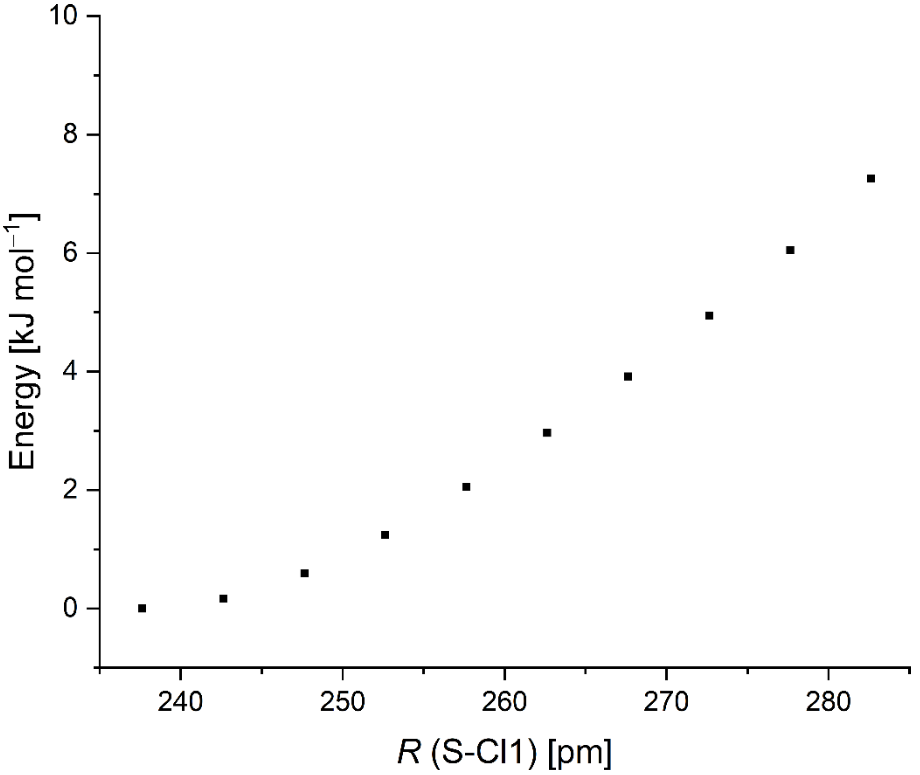

Figure S 11. Relaxed surface scan for $[SCl_6]^{2-}$ (part).

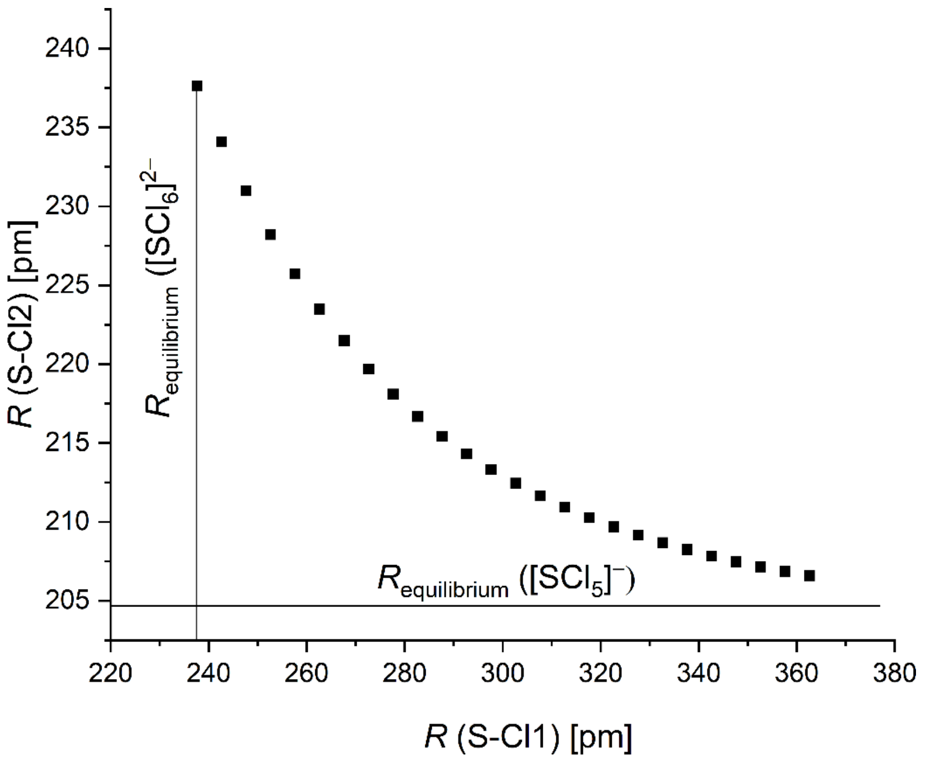

Figure S 12. Correlation between R(S-Cl2) and R(S-Cl1).

h) Solid State Calculations

Periodic density functional theory (DFT) calculations with the B3LYP functional and cc-pVDZ basis set[16] were performed with the CRYSTAL17 program[17]. Electron dispersion effects were included via the empirical D3 scheme[11,18]. The first Brillouin zone was sampled using an 6×6×6 Monkhorst-Pack grid. To facilitate convergence, the Coulomb and exchange integral thresholds were sufficiently tightened with the TOLINTEG keyword to values of 8, 8, 8, 16 and 32. AIM analysis was performed with the TOPOND code developed by Gatti[19] and recently implemented in CRYSTAL17. The estimated stabilization energy for one $[SCl_6]^{2-}$ molecule in the solid was calculated as

$$\Delta E = 0.5 \cdot (E_{bulk} - 2 \cdot E_{dianion} - E_{cation\text{-}lattice}),$$

where E_{bulk} is the total energy of the periodic $[NEt_3Me]_2[SCl_6]$ per unit cell, $E_{dianion}$ is the total energy of one isolated $[SCl_6]^{2-}$ ion, and $E_{cation\text{-}lattice}$ the total energy of the cation sub-lattice per unit cell. All three energies were calculated based on the experimental crystal structure.

Table S 5. Properties for all 52 symmetry-inequivalent bond critical points (BCP) found in the unit cell of the periodic $[NEt_3Me]_2[SCl_6]$ structure. Columns A1 and A2 list the two bound atoms; r_{A1-A2} the bond distance; ρ_{BCP}, $\nabla^2\rho_{BCP}$, ELF_{BCP} the values of the electron density, Laplacian of the electron density and electron localization function at the BCP, and G_{BCP}, V_{BCP}, $|V_{BCP}|/G_{BCP}$ the values for the potential and kinetic energy density as well as there ratio at the BCP.

#	A1	A2	r_{A1-A2} [Å]	ρ_{BCP} [e/Å^3]	$\nabla^2\rho_{BCP}$ [e/Å^5]	ELF_{BCP}	G_{BCP} a.u.	V_{BCP} a.u.	$\|V_{BCP}\|/G_{BCP}$
**1**	1 S	7 Cl	2.3120	0.584	1.737	0.556	4.3341E-02	-6.8662E-02	1.58
**2**	1 S	3 Cl	2.3153	0.580	1.750	0.555	4.3064E-02	-6.7972E-02	1.58
**3**	1 S	11 Cl	2.3232	0.571	1.786	0.550	4.2302E-02	-6.6079E-02	1.56
**5**	2 Cl	66 H	2.7936	0.056	0.633	0.028	5.6361E-03	-4.7054E-03	0.83
**6**	2 Cl	54 H	2.9112	0.046	0.506	0.025	4.5013E-03	-3.7515E-03	0.83
**7**	2 Cl	68 H	3.0730	0.036	0.432	0.016	3.6537E-03	-2.8247E-03	0.77
**11**	3 Cl	55 H	2.7364	0.065	0.724	0.035	6.5395E-03	-5.5720E-03	0.85
**12**	3 Cl	88 H	2.8692	0.048	0.511	0.027	4.5879E-03	-3.8780E-03	0.85
**13**	3 Cl	68 H	2.9875	0.041	0.444	0.020	3.9400E-03	-3.2785E-03	0.83
**14**	3 Cl	107 H	3.0003	0.045	0.564	0.021	4.6468E-03	-3.4448E-03	0.74
**15**	3 Cl	60 H	3.0308	0.037	0.412	0.019	3.5849E-03	-2.9001E-03	0.81
**16**	3 Cl	52 H	3.1388	0.025	0.310	0.010	2.6051E-03	-1.9952E-03	0.77
**17**	4 Cl	68 H	2.7983	0.058	0.638	0.031	5.7683E-03	-4.9194E-03	0.85
**18**	4 Cl	47 H	2.8678	0.049	0.537	0.027	4.7975E-03	-4.0211E-03	0.84
**23**	5 N	35 C	1.5005	1.603	-11.841	0.806	1.2820E-01	-3.7924E-01	2.96
**24**	5 N	19 C	1.5144	1.556	-11.103	0.809	1.2105E-01	-3.5729E-01	2.95
**25**	5 N	27 C	1.5195	1.538	-10.738	0.806	1.1974E-01	-3.5088E-01	2.93
**26**	5 N	23 C	1.5210	1.536	-10.704	0.811	1.1774E-01	-3.4653E-01	2.94
**27**	6 C	47 H	0.9900	2.344	-38.054	0.986	5.7850E-02	-5.1048E-01	8.82
**28**	6 C	51 H	0.9900	2.351	-38.377	0.988	5.5288E-02	-5.0869E-01	9.20
**29**	6 C	39 C	1.5161	1.656	-12.626	0.950	6.3120E-02	-2.5722E-01	4.08
**30**	7 C	55 H	0.9900	2.353	-38.375	0.987	5.5826E-02	-5.0974E-01	9.13
**31**	7 C	59 H	0.9900	2.344	-38.165	0.987	5.7015E-02	-5.0995E-01	8.94
**32**	7 C	31 C	1.5160	1.656	-12.621	0.950	6.3150E-02	-2.5723E-01	4.07
**33**	8 C	63 H	0.9900	2.344	-38.025	0.986	5.8321E-02	-5.1112E-01	8.76
**34**	8 C	67 H	0.9880	2.356	-38.621	0.988	5.3666E-02	-5.0798E-01	9.47

#	A1	A2	r_{A1-A2} [Å]	ρ_{BCP} [e/Å^3]	$\nabla^2\rho_{BCP}$ [e/Å^5]	ELF_{BCP}	G_{BCP} a.u.	V_{BCP} a.u.	$\lvert V_{BCP}\rvert/G_{BCP}$
**35**	8 C	43 C	1.5136	1.664	-12.725	0.951	6.3510E-02	-2.5903E-01	4.08
**36**	9 C	71 H	0.9800	2.318	-36.835	0.977	7.4074E-02	-5.3027E-01	7.16
**37**	9 C	75 H	0.9800	2.319	-12.750	0.978	7.3145E-02	-5.2909E-01	7.23
**38**	9 C	79 H	0.9800	2.299	-36.401	0.976	7.5138E-02	-5.2790E-01	7.03
**39**	9 C	15 N	2.5579	0.071	1.343	0.019	1.0485E-02	-7.0415E-03	0.67
**40**	10 C	87 H	0.9800	2.364	-38.806	0.986	6.0136E-02	-5.2285E-01	8.69
**41**	11 C	95 H	0.9760	2.314	-36.674	0.976	7.5541E-02	-5.3253E-01	7.05
**42**	12 C	115 H	0.9760	2.316	-36.731	0.976	7.5297E-02	-5.3164E-01	7.06
**44**	15 H	83 H	2.2872	0.021	0.278	0.008	2.1638E-03	-1.4457E-03	0.67
**45**	16 H	104 H	2.4182	0.028	0.406	0.009	3.2553E-03	-2.2958E-03	0.71
**46**	17 H	75 H	2.1191	0.071	1.204	0.022	9.7410E-03	-6.9932E-03	0.72
**52**	24 H	107 H	2.1834	0.016	0.210	0.006	1.6042E-03	-1.0255E-03	0.64

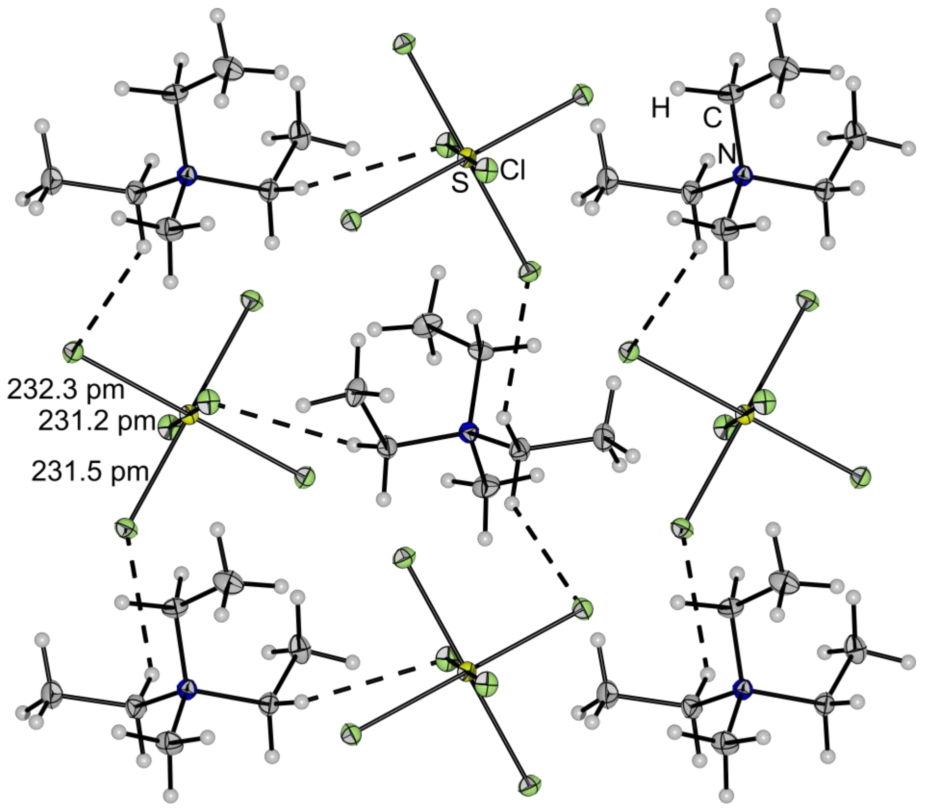

Figure S 13. Molecular structure in the solid state of $[NEt_3Me]_2[SCl_6]$ for comparison to the periodic calculation.

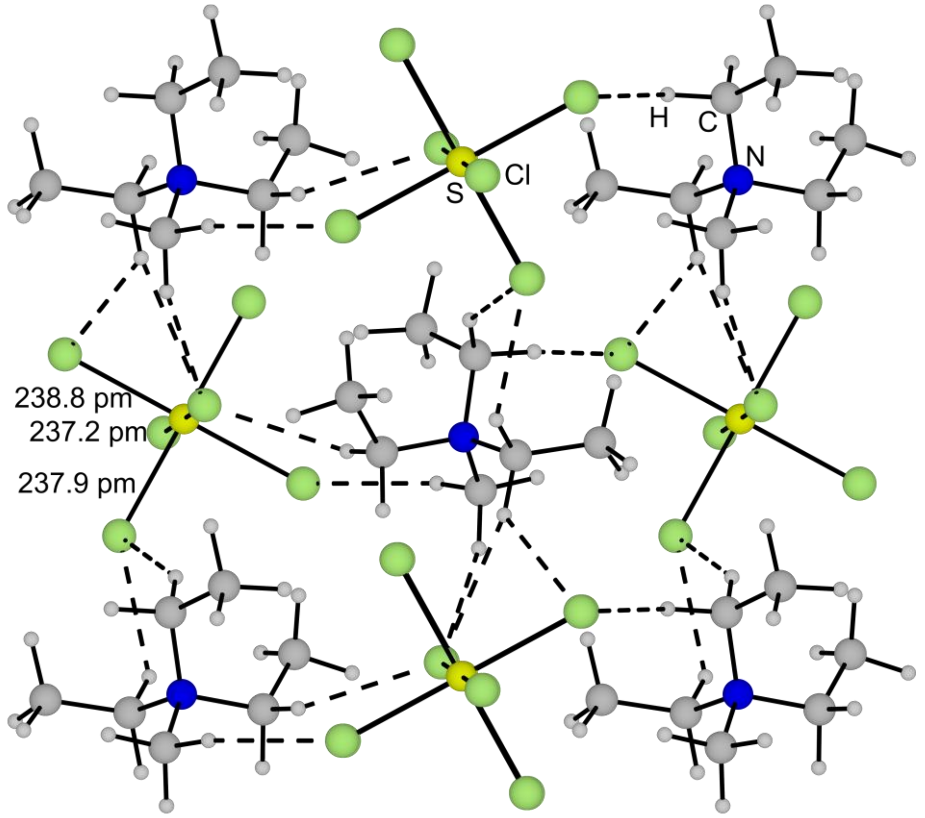

Figure S 14. Optimized structure using a periodic model for $[NEt_3Me]_2[SCl_6]$

i) Optimized Structures

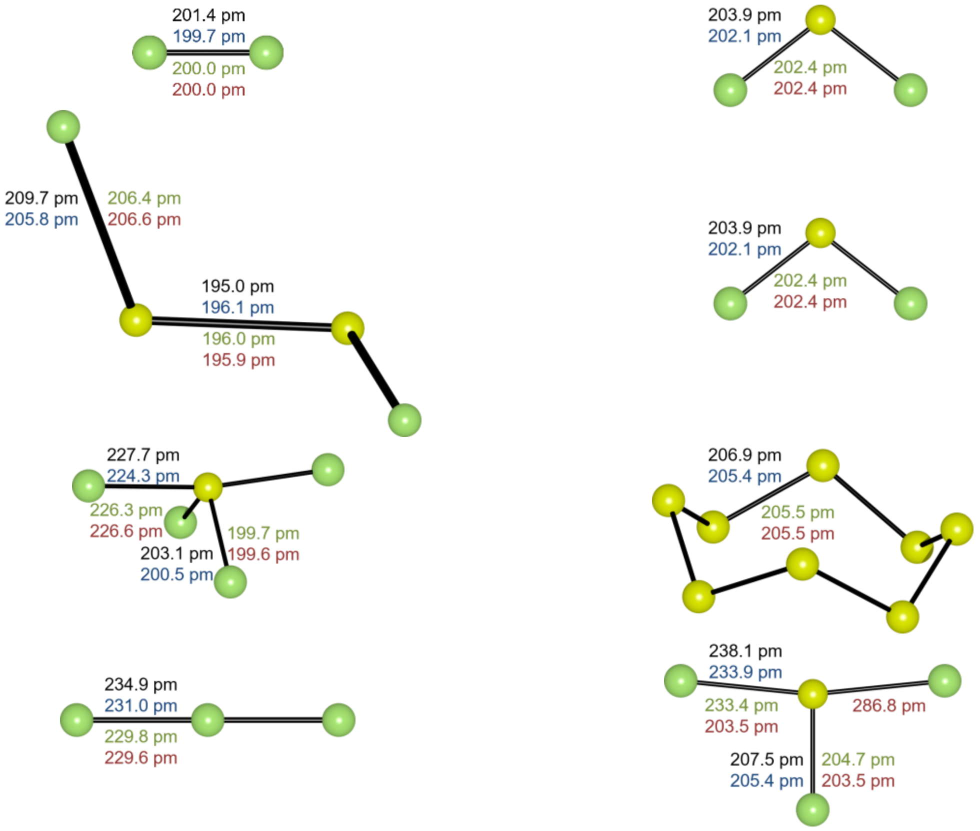

Figure S 15. Optimized structures calculated on the B3LYP(D4)/def2-TZVPP (black), SCS-MP2/def2-TZVPP (blue), Cosmo-SCS-MP2/def2-TZVPP with (εr = 10) (green) and Cosmo-SCS-MP2/def2-TZVPP with (εr = 100) (red) level of theory.

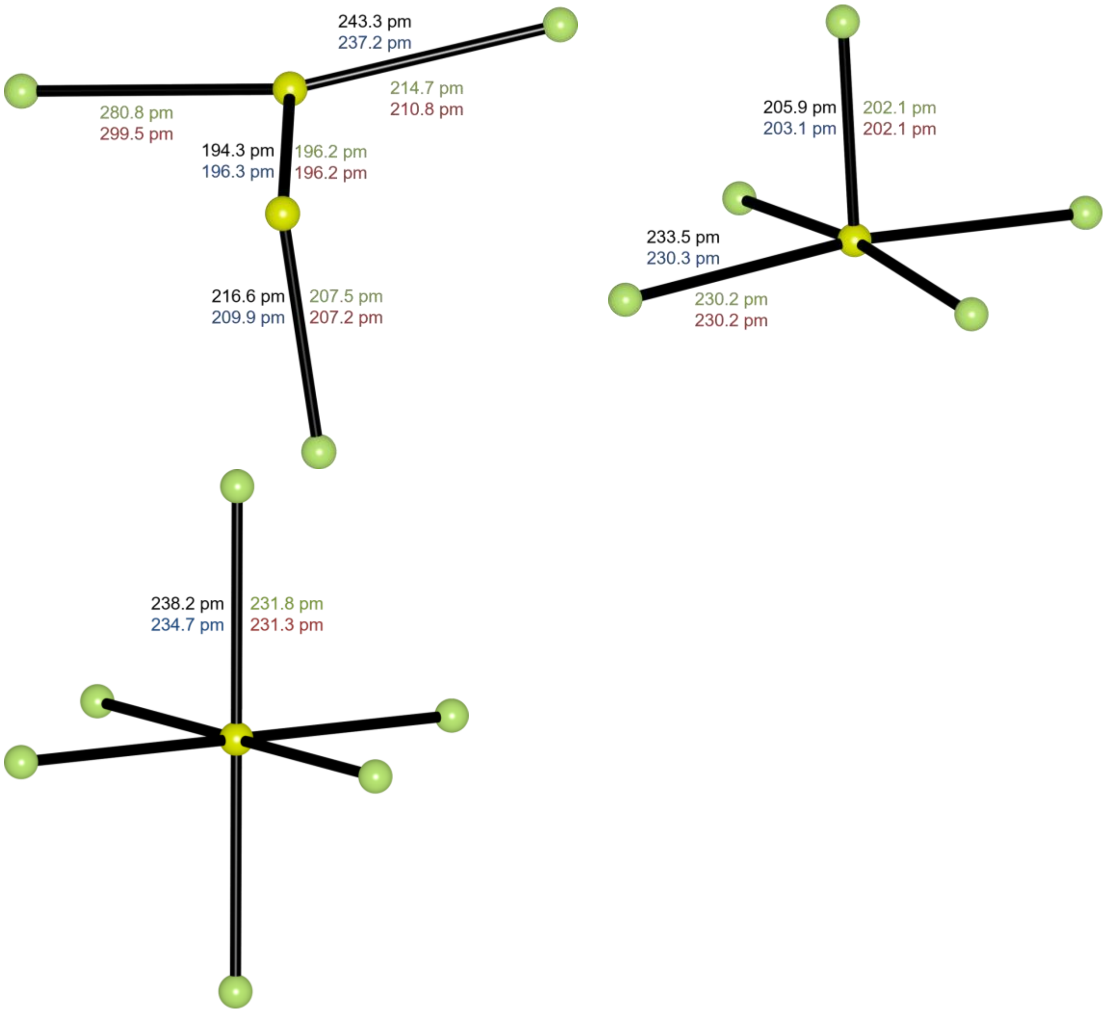

Figure S 16. Optimized structures calculated on the B3LYP(D4)/def2-TZVPP (black), SCS-MP2/def2-TZVPP (blue), Cosmo-SCS-MP2/def2-TZVPP with (ε_r = 10) (green) and Cosmo-SCS-MP2/def2-TZVPP with (ε_r = 100) (red) level of theory.

j) Calculated Energies and Free Reaction Energies

j1) B3LYP(D4)/def2-TZVPP Energies

All Free Energy calculations were carried out for T = 298.15 K and p = 1.0 bar.

Table S 6. Calculated energies on B3LYP(D4)/def2-TZVPP level of theory.

Compound	E_{tot} / E_H	E_{tot} / kJ mol^{-1}	G / kJ mol^{-1}
Cl^-	–460.22009153	–1208307.9	–1208347.3
Cl_2	–920.29295228	–2416229.1	–2416283.4
$[Cl_3]^-$	–1380.56911826	–3624684.2	–3624747.3
$[Cl_5]^-$	–2300.88506425	–6040973.7	–6041061.9
S_8	–3185.38469	–8363227.5	–8363292.6
S_2Cl_2	–1716.66099442	–4507093.4	–4507161.5
SCl_2	–1318.47707733	–3461661.6	–3461726.2
SCl_4	–2238.77338942	–5877899.5	–5877970.8
$[S_2Cl_3]^-$	–2176.934085	–5715540.4	–5715620.6
$[SCl_3]^-$	–1778.74899354	–4670105.5	–4670181.2
$[SCl_5]^-$	–2699.06736670	–7086401.4	–7086478.6
$[SCl_6]^{2-}$	–3159.21730437	–8294525.0	–8294606.0

j2) SCS-MP2/def2-TZVPP Energies

Table S 7. Calculated energies on SCS-MP2/def2-TZVPP level of theory.

Compound	E_{tot} / E_H	E_{MP2} / E_H	$E_{tot+MP2}$ / kJ mol^{-1}	G / kJ mol^{-1}
Cl^-	–459.55562242	–0.346212077	–1207472.27	–1207511.75
Cl_2	–918.98287991	–0.67866770	–2414571.39	–2414625.42
$[Cl_3]^-$	–1378.55619762	–1.04983132	–3622155.63	–3622217.50
$[Cl_5]^-$	–2297.54169011	–1.73983938	–6036763.66	–6036852.28
S_8	–3180.36529468	–2.53454576	–8356703.53	–8356765.52
S_2Cl_2	–1714.09391978	–1.31117234	–4503796.07	–4503862.97
SCl_2	–1316.54256107	–0.99272865	–3459188.90	–3459252.84
SCl_4	–2235.48081451	–1.70958138	–5873743.38	–5873811.61
$[S_2Cl_3]^-$	–2173.66974220	–1.67968992	–5711379.93	–5711457.62
$[SCl_3]^-$	–1776.11860560	–1.36104112	–4666772.81	–4666846.56
$[SCl_5]^-$	–2695.06746152	–2.09289076	–7081394.50	–7081467.87
$[SCl_6]^{2-}$	–3154.51139576	–2.47971458	–8288680.16	–8288752.86

Table S 8. Calculated energies on Cosmo-SCS-MP2/def2-TZVPP (ε_r = 10) level of theory.

Compound	E_{tot} / E_H	E_{MP2} / E_H	$E_{tot+MP2}$ / kJ mol^{-1}	G / kJ mol^{-1}
Cl^-	–459.6633456	–0.34613543	–1207754.893	–1207794.381
Cl_2	–918.9842183	–0.678549984	–2414574.598	–2414628.688
$[Cl_3]^-$	–1378.62918	–1.049801827	–3622347.166	–3622410.036
$[Cl_5]^-$	–2297.622704	–1.72535081	–6036938.319	–6037030.549
S_8	–3180.368375	–2.533420838	–8356708.665	–8356771.545
S_2Cl_2	–1714.096259	–1.310756176	–4503801.119	–4503868.529
SCl_2	–1316.544514	–0.992405961	–3459193.182	–3459257.372
SCl_4	–2235.489494	–1.707635175	–5873761.061	–5873830.591
$[S_2Cl_3]^-$	–2173.752312	–1.668086776	–5711566.256	–5711647.546
$[SCl_3]^-$	–1776.191788	–1.360170229	–4666962.667	–4667038.997
$[SCl_5]^-$	–2695.136970	–2.091107879	–7081572.319	–7081646.659
$[SCl_6]^{2-}$	–3154.752235	–2.480096521	–8289313.487	–8289388.007

Table S 9. Calculated energies on Cosmo-SCS-MP2/def2-TZVPP (ε_r = 100) level of theory.

Compound	E_{tot} / E_H	E_{MP2} / E_H	$E_{tot+MP2}$ / kJ mol^{-1}	G / kJ mol^{-1}
Cl^-	–459.6794238	–0.346124185	–1207797.076	–1207836.565
Cl_2	–918.9844248	–0.67853317	–2414575.096	–2414629.196
$[Cl_3]^-$	–1378.642319	–1.049811384	–3622381.689	–3622445.039
$[Cl_5]^-$	–2297.638332	–1.721391767	–6036968.955	–6037061.055
S_8	–3180.368853	–2.533246899	–8356709.463	–8356772.383
S_2Cl_2	–1714.096651	–1.310697273	–4503801.994	–4503869.474
SCl_2	–1316.544829	–0.992352347	–3459193.87	–3459258.09
SCl_4	–2235.491051	–1.707287039	–5873764.236	–5873834.086
$[S_2Cl_3]^-$	–2173.769121	–1.664525301	–5711601.039	–5711682.859
$[SCl_3]^-$	–1776.217233	–1.347866492	–4666997.17	–4667074.33
$[SCl_5]^-$	–2695.147504	–2.090806612	–7081599.185	–7081673.785
$[SCl_6]^{2-}$	–3154.788322	–2.480188857	–8289408.476	–8289482.736

j3) Free Reaction Energy Calculation

Table S 10. Δ*E* for the reactions between sulfur and chlorine containing compounds calculated on the B3LYP(D4)/def2-TZVPP, SCS-MP2/def2-TZVPP, Cosmo-SCS-MP2/def2-TZVPP with (ε_r = 10) (Cosmo1) and Cosmo-SCS-MP2/def2-TZVPP with (ε_r = 100) (Cosmo2) level of theory.

Reaction	ΔE_{B3LYP} / kJ mol^{-1}	ΔE_{MP2} / kJ mol^{-1}	$\Delta E_{Cosmo1\text{-}MP2}$ / kJ mol^{-1}	$\Delta E_{Cosmo2\text{-}MP2}$ / kJ mol^{-1}
¼ S_8 + Cl_2 → S_2Cl_2	–57.4	–48.8	–49.4	–49.5
⅛ S_8 + Cl_2 → SCl_2	–29.0	0.0	–30.0	–30.1
⅛ S_8 + 2 Cl_2 → SCl_4	–37.8	–12.7	–23.3	–25.4
S_2Cl_2 + Cl_2 → 2 SCl_2	–0.5	–10.3	–10.6	–10.6
SCl_2 + Cl_2 → 2 SCl_4	–8.8	16.9	6.7	4.7
¼ S_8 + $[Cl_3]^-$ → $[S_2Cl_3]^-$	–49.3	–48.4	–41.9	–42.0
⅛ S_8 + $[Cl_3]^-$ → $[SCl_3]^-$	–17.8	–29.2	–26.9	–26.8
⅛ S_8 + $[Cl_5]^-$ → $[SCl_5]^-$	–24.2	–42.9	–45.4	–41.5
$[S_2Cl_3]^-$ + $[Cl_3]^-$ → 2 $[SCl_3]^-$	13.7	–10.1	–11.9	–11.6
$[SCl_3]^-$ + Cl_2 → $[SCl_5]^-$	–66.7	–50.3	–35.1	–26.9

Table S 11. Δ*G* for the reactions between sulfur and chlorine containing compounds calculated on the B3LYP(D4)/def2-TZVPP, SCS-MP2/def2-TZVPP, Cosmo-SCS-MP2/def2-TZVPP with (ε_r = 10) (Cosmo1) and Cosmo-SCS-MP2/def2-TZVPP with (ε_r = 100) (Cosmo2) level of theory.

Reaction	ΔG_{B3LYP} / kJ mol^{-1}	ΔG_{MP2} / kJ mol^{-1}	$\Delta G_{Cosmo1\text{-}MP2}$ / kJ mol^{-1}	$\Delta G_{Cosmo2\text{-}MP2}$ / kJ mol^{-1}
¼ S_8 + Cl_2 → S_2Cl_2	–55.0	–46.2	–47.0	–47.2
⅛ S_8 + Cl_2 → SCl_2	–31.2	–31.7	–32.2	–32.3
⅛ S_8 + 2 Cl_2 → SCl_4	7.6	34.9	23.2	20.9
S_2Cl_2 + Cl_2 → 2 SCl_2	–7.5	–17.3	–17.5	–17.5
SCl_2 + Cl_2 → 2 SCl_4	38.8	66.7	55.5	53.2
¼ S_8 + $[Cl_3]^-$ → $[S_2Cl_3]^-$	–50.1	–48.7	–44.6	–44.7
⅛ S_8 + $[Cl_3]^-$ → $[SCl_3]^-$	–22.4	–33.4	–32.5	–32.7
⅛ S_8 + $[Cl_5]^-$ → $[SCl_5]^-$	–5.1	–19.9	–19.7	–16.2
$[S_2Cl_3]^-$ + $[Cl_3]^-$ → 2 $[SCl_3]^-$	5.4	–18.0	–20.4	–20.8
$[SCl_3]^-$ + Cl_2 → $[SCl_5]^-$	–14.0	4.1	21.0	29.7

k) Calculated Vibrational Frequencies

Table S 12. Cl_2

B3LYP(D4)/def2-TZVPP				SCS-MP2/def2-TZVPP			
Nr.	Symmetry	Wavenumber / cm^{-1}	IR intensity / km mol^{-1}	Nr.	Symmetry	Wavenumber / cm^{-1}	IR intensity / km mol^{-1}
1	Σ_g^+	537.0	0	1	Σ_g^+	560.6	0

SCS-MP2/def2-TZVPP-Cosmo (ε_r = 10)				SCS-MP2/def2-TZVPP-Cosmo (ε_r = 100)			
Nr.	Symmetry	Wavenumber / cm^{-1}	IR intensity / km mol^{-1}	Nr.	Symmetry	Wavenumber / cm^{-1}	IR intensity / km mol^{-1}
1	Σ_g^+	557.0	0	1	Σ_g^+	556.4	0

Table S 13. $[Cl_3]^-$

B3LYP(D4)/def2-TZVPP				SCS-MP2/def2-TZVPP			
Nr.	Symmetry	Wavenumber / cm^{-1}	IR intensity / km mol^{-1}	Nr.	Symmetry	Wavenumber / cm^{-1}	IR intensity / km mol^{-1}
1	Π_u	154.5	1	1	Π_u	167.9	1
2	Π_u	154.5	1	2	Π_u	167.9	1
3	Σ_g^+	247.3	0	3	Σ_g^+	270.5	0
4	Σ_u^-	261.6	410	4	Σ_u^-	295.1	569

SCS-MP2/def2-TZVPP-Cosmo (ε_r = 10)				SCS-MP2/def2-TZVPP-Cosmo (ε_r = 100)			
Nr.	Symmetry	Wavenumber / cm^{-1}	IR intensity / km mol^{-1}	Nr.	Symmetry	Wavenumber / cm^{-1}	IR intensity / km mol^{-1}
1	Π_u	168.8	1	1	Π_u	168.5	1
2	Π_u	168.8	1	2	Π_u	168.5	1
3	Σ_u^-	202.8	1392	3	Σ_u^-	168.7	1657
4	Σ_g^+	277.8	0	4	Σ_g^+	278.9	0

Table S 14. $[Cl(Cl_2)_2]^-$

B3LYP(D4)/def2-TZVPP				SCS-MP2/def2-TZVPP			
Nr.	Symmetry[1]	Wavenumber / cm^{-1}	IR intensity / km mol^{-1}	Nr.	Symmetry	Wavenumber / cm^{-1}	IR intensity / km mol^{-1}
1	*A*	18.8	0	1	*A*	17.7	0
2	*A*	128.3	33	2	*A*	92.4	743
3	*A*	134.9	0	3	*A*	140.0	17
4	*A*	135.9	7	4	*A*	143.4	0
5	*A*	148.8	1	5	*A*	152.2	192
6	*A*	187.8	452	6	*A*	160.4	1
7	*A*	200.5	18	7	*A*	203.3	45
8	*A*	304.3	376	8	*A*	326.3	270
9	*A*	353.0	91	9	*A*	374.5	125

SCS-MP2/def2-TZVPP-Cosmo (ε_r = 10)				SCS-MP2/def2-TZVPP-Cosmo (ε_r = 100)			
Nr.	Symmetry	Wavenumber / cm^{-1}	IR intensity / km mol^{-1}	Nr.	Symmetry	Wavenumber / cm^{-1}	IR intensity / km mol^{-1}
1	*A*	17.1	2	1	*A*	18.2	3
2	*A*	58.4	223	2	*A*	74.1	105
3	*A*	104.5	99	3	*A*	97.4	81
4	*A*	118.0	0	4	*A*	108.7	0
5	*A*	124.8	35	5	*A*	116.5	36
6	*A*	133.0	4	6	*A*	120.4	10
7	*A*	135.8	57	7	*A*	122.5	41
8	*A*	419.0	232	8	*A*	457.9	143
9	*A*	440.5	162	9	*A*	469.9	121

[1] $[Cl(Cl_2)_2]^-$ was optimized in C_1 symmetries because conversion issues for Cosmo calculations arise at higher symmetries

Table S 15. S_8

B3LYP(D4)/def2-TZVPP				SCS-MP2/def2-TZVPP			
Nr.	Symmetry	Wavenumber / cm^{-1}	IR intensity / km mol^{-1}	Nr.	Symmetry	Wavenumber / cm^{-1}	IR intensity / km mol^{-1}
1	E_2	68.9	0	1	E_2	68.0	0
2	E_2	68.9	0	2	E_2	68.0	0
3	E_2	146.0	0	3	E_2	154.0	0
4	E_2	146.0	0	4	E_2	154.0	0
5	E_1	191.2	4	5	E_1	195.2	3
6	E_1	191.2	4	6	E_1	195.2	3
7	A_1	215.1	0	7	A_1	219.0	0
8	B_2	243.2	3	8	E_3	252.3	0
9	E_3	248.4	0	9	E_3	252.3	0
10	E_3	248.4	0	10	B_2	253.2	4
11	B_1	389.8	0	11	B_1	452.4	0
12	E_3	415.7	0	12	E_3	465.6	0
13	E_3	415.7	0	13	E_3	465.6	0
14	E_2	461.8	0	14	E_1	485.8	1
15	E_2	461.8	0	15	E_1	485.8	1
16	E_1	466.8	2	16	E_2	489.7	0
17	E_1	466.8	2	17	E_2	489.7	0
18	A_1	475.3	0	18	A_1	490.2	0

SCS-MP2/def2-TZVPP-Cosmo (ε_r = 10)				SCS-MP2/def2-TZVPP-Cosmo (ε_r = 100)			
Nr.	Symmetry	Wavenumber / cm^{-1}	IR intensity / km mol^{-1}	Nr.	Symmetry	Wavenumber / cm^{-1}	IR intensity / km mol^{-1}
1	E_2	65.8	0	1	E_2	65.7	0
2	E_2	65.8	0	2	E_2	65.7	0
3	E_2	153.2	0	3	E_2	153.1	0
4	E_2	153.2	0	4	E_2	153.1	0
5	E_1	193.4	7	5	E_1	193.1	8
6	E_1	193.4	7	6	E_1	193.1	8
7	A_1	217.2	0	7	A_1	216.8	0
8	E_3	249.9	0	8	E_3	249.4	0
9	E_3	249.9	0	9	E_3	249.4	0
10	B_2	251.2	7	10	B_2	250.8	8
11	B_1	453.0	0	11	B_1	453.1	0
12	E_3	465.2	0	12	E_3	465.2	0
13	E_3	465.2	0	13	E_3	465.2	0
14	E_1	483.8	2	14	E_1	483.5	3
15	E_1	483.8	2	15	E_1	483.5	3
16	A_1	487.1	0	16	A_1	486.6	0
17	E_2	488.2	0	17	E_2	488.0	0
18	E_2	488.2	0	18	E_2	488.0	0

Table S 16. S_2Cl_2

B3LYP(D4)/def2-TZVPP				SCS-MP2/def2-TZVPP			
Nr.	Symmetry	Wavenumber / cm^{-1}	IR intensity / km mol^{-1}	Nr.	Symmetry	Wavenumber / cm^{-1}	IR intensity / km mol^{-1}
1	*A*	93.6	0	1	*A*	95.5	0
2	*A*	205.2	1	2	*A*	208.7	0
3	*B*	236.3	7	3	*B*	247.5	5
4	*B*	424.4	129	4	*B*	476.3	92
5	*A*	438.9	38	5	*A*	487.1	33
6	*A*	545.2	7	6	*A*	537.9	5

SCS-MP2/def2-TZVPP-Cosmo (ε_r = 10)				SCS-MP2/def2-TZVPP-Cosmo (ε_r = 100)			
Nr.	Symmetry	Wavenumber / cm^{-1}	IR intensity / km mol^{-1}	Nr.	Symmetry	Wavenumber / cm^{-1}	IR intensity / km mol^{-1}
1	*A*	94.6	0	1	*A*	94.4	0
2	*A*	208.0	0	2	*A*	207.8	0
3	*B*	244.3	12	3	*B*	243.7	14
4	*B*	458.1	163	4	*B*	454.2	182
5	*A*	471.4	65	5	*A*	467.8	73
6	*A*	534.5	6	6	*A*	534.2	6

Table S 17. SCl_2

B3LYP(D4)/def2-TZVPP				SCS-MP2/def2-TZVPP			
Nr.	Symmetry	Wavenumber / cm^{-1}	IR intensity / km mol^{-1}	Nr.	Symmetry	Wavenumber / cm^{-1}	IR intensity / km mol^{-1}
1	A_1	199.6	0	1	A_1	209.9	0
2	B_1	493.9	77	2	B_1	531.0	65
3	A_1	508.2	10	3	A_1	532.8	11

SCS-MP2/def2-TZVPP-Cosmo (ε_r = 10)				SCS-MP2/def2-TZVPP-Cosmo (ε_r = 100)			
Nr.	Symmetry	Wavenumber / cm^{-1}	IR intensity / km mol^{-1}	Nr.	Symmetry	Wavenumber / cm^{-1}	IR intensity / km mol^{-1}
1	A_1	208.7	1	1	A_1	208.6	1
2	B_1	517.3	110	2	B_1	514.7	121
3	A_1	526.6	20	3	A_1	525.6	21

Table S 18. SCl_4

B3LYP(D4)/def2-TZVPP				SCS-MP2/def2-TZVPP			
Nr.	Symmetry	Wavenumber / cm^{-1}	IR intensity / km mol^{-1}	Nr.	Symmetry	Wavenumber / cm^{-1}	IR intensity / km mol^{-1}
1	A_1	100.4	2	1	A_1	114.5	3
2	B_2	151.2	2	2	B_2	175.0	3
3	A_2	187.2	0	3	A_2	208.3	0
4	B_1	225.0	13	4	B_1	246.3	8
5	A_1	228.0	0	5	A_1	254.0	0
6	A_1	256.4	6	6	A_1	272.4	4
7	B_1	370.8	521	7	B_1	416.5	536
8	B_2	479.7	105	8	A_1	520.3	27
9	A_1	484.4	31	9	B_2	529.6	92

SCS-MP2/def2-TZVPP-Cosmo (ε_r = 10)				SCS-MP2/def2-TZVPP-Cosmo (ε_r = 100)			
Nr.	Symmetry	Wavenumber / cm^{-1}	IR intensity / km mol^{-1}	Nr.	Symmetry	Wavenumber / cm^{-1}	IR intensity / km mol^{-1}
1	A_1	115.6	5	1	A_1	115.4	6
2	B_2	171.9	7	2	B_2	171.2	8
3	A_2	204.8	0	3	A_2	204.1	0
4	B_1	239.3	85	4	B_1	235.7	186
5	A_1	250.8	1	5	A_1	250.1	1
6	A_1	264.9	8	6	A_1	263.6	9
7	B_1	329.1	1153	7	B_1	306.4	1270
8	A_1	519.8	47	8	A_1	519.4	52
9	B_2	522.6	165	9	B_2	521.0	183

Table S 19. $[S_2Cl_3]^–$

B3LYP(D4)/def2-TZVPP				SCS-MP2/def2-TZVPP			
Nr.	Symmetry[1]	Wavenumber / cm$^{–1}$	IR intensity / km mol$^{–1}$	Nr.	Symmetry	Wavenumber / cm$^{–1}$	IR intensity / km mol$^{–1}$
1	*A*	65.8	1	1	*A*	64.7	2
2	*A*	80.4	1	2	*A*	96.9	1
3	*A*	130.5	6	3	*A*	149.9	6
4	*A*	185.2	71	4	*A*	200.0	82
5	*A*	207.7	9	5	*A*	237.8	2
6	*A*	226.2	25	6	*A*	244.0	19
7	*A*	258.8	373	7	*A*	270.0	553
8	*A*	369.8	109	8	*A*	428.0	92
9	*A*	545.0	5	9	*A*	524.3	2

SCS-MP2/def2-TZVPP-Cosmo (ε_r = 10)				SCS-MP2/def2-TZVPP-Cosmo (ε_r = 100)			
Nr.	Symmetry	Wavenumber / cm$^{–1}$	IR intensity / km mol$^{–1}$	Nr.	Symmetry	Wavenumber / cm$^{–1}$	IR intensity / km mol$^{–1}$
1	*A*	51.4	29	1	*A*	44.8	12
2	*A*	79.4	207	2	*A*	79.8	92
3	*A*	92.4	9	3	*A*	84.1	13
4	*A*	111.8	11	4	*A*	99.7	7
5	*A*	214.6	1	5	*A*	212.8	1
6	*A*	242.4	14	6	*A*	242.3	14
7	*A*	345.5	359	7	*A*	397.5	279
8	*A*	451.0	120	8	*A*	454.1	123
9	*A*	521.6	2	9	*A*	524.9	2

[1] $[S_2Cl_3]^–$ was optimized in C_1 symmetries because conversion issues for Cosmo calculations arise at higher symmetries

Table S 20. $[SCl_3]^-$

B3LYP(D4)/def2-TZVPP				SCS-MP2/def2-TZVPP			
Nr.	Symmetry[1]	Wavenumber / cm^{-1}	IR intensity / km mol^{-1}	Nr.	Symmetry	Wavenumber / cm^{-1}	IR intensity / km mol^{-1}
1	*A*	106.5	2	1	*A*	129.3	2
2	*A*	156.9	44	2	*A*	175.4	61
3	*A*	175.0	2	3	*A*	186.1	2
4	*A*	231.0	5	4	*A*	256.7	3
5	*A*	261.3	447	5	*A*	278.2	604
6	*A*	466.3	40	6	*A*	501.5	33

SCS-MP2/def2-TZVPP-Cosmo (ε_r = 10)				SCS-MP2/def2-TZVPP-Cosmo (ε_r = 100)			
Nr.	Symmetry	Wavenumber / cm^{-1}	IR intensity / km mol^{-1}	Nr.	Symmetry	Wavenumber / cm^{-1}	IR intensity / km mol^{-1}
1	*A*	92.1	1256	1	*A*	78.3	99
2	*A*	134.4	5	2	*A*	94.8	62
3	*A*	182.5	4	3	*A*	130.5	4
4	*A*	199.8	147	4	*A*	206.2	8
5	*A*	258.6	4	5	*A*	426.1	289
6	*A*	504.0	50	6	*A*	512.0	57

[1] $[SCl_3]^-$ was optimized in C_1 symmetries because conversion issues for Cosmo calculations arise at higher symmetries

Table S 21. $[SCl_5]^-$

B3LYP(D4)/def2-TZVPP				SCS-MP2/def2-TZVPP			
Nr.	Symmetry	Wavenumber / cm^{-1}	IR intensity / km mol^{-1}	Nr.	Symmetry	Wavenumber / cm^{-1}	IR intensity / km mol^{-1}
1	B_2	79.0	0	1	B_2	94.4	0
2	E	128.3	11	2	E	137.7	6
3	E	128.3	11	3	E	137.7	6
4	B_1	160.5	0	4	B_1	163.3	0
5	A_1	162.7	1	5	A_1	187.4	3
6	E	186.3	38	6	E	205.4	23
7	E	186.3	38	7	E	205.4	23
8	B_2	214.7	0	8	B_2	238.8	0
9	A_1	252.7	7	9	A_1	264.9	3
10	E	348.2	442	10	E	407.2	457
11	E	348.2	442	11	E	407.2	457
12	A_1	459.9	62	12	A_1	503.1	53

SCS-MP2/def2-TZVPP-Cosmo (ε_r = 10)				SCS-MP2/def2-TZVPP-Cosmo (ε_r = 100)			
Nr.	Symmetry	Wavenumber / cm^{-1}	IR intensity / km mol^{-1}	Nr.	Symmetry	Wavenumber / cm^{-1}	IR intensity / km mol^{-1}
1	B_2	97.2	0	1	B_2	97.5	0
2	E	136.7	18	2	E	136.2	26
3	E	136.7	18	3	E	136.2	26
4	B_1	163.0	0	4	B_1	162.9	0
5	A_1	189.4	9	5	A_1	189.3	11
6	E	205.4	78	6	E	204.5	108
7	E	205.4	78	7	E	204.5	108
8	B_2	242.6	0	8	B_2	243.2	0
9	A_1	266.7	5	9	A_1	267.0	6
10	E	357.5	1016	10	E	343.9	1177
11	E	357.5	1016	11	E	343.9	1177
12	A_1	504.5	88	12	A_1	504.5	97

Table S 22. $[SCl_6]^{2-}$

B3LYP(D4)/def2-TZVPP				SCS-MP2/def2-TZVPP			
Nr.	Symmetry	Wavenumber / cm^{-1}	IR intensity / km mol^{-1}	Nr.	Symmetry	Wavenumber / cm^{-1}	IR intensity / km mol^{-1}
1	T_{2u}	103.6	0	1	T_{2u}	112.5	0
2	T_{2u}	103.6	0	2	T_{2u}	112.5	0
3	T_{2u}	103.6	0	3	T_{2u}	112.5	0
4	T_{1u}	127.9	82	4	T_{2g}	151.2	0
5	T_{1u}	127.9	82	5	T_{2g}	151.2	0
6	T_{1u}	127.9	82	6	T_{2g}	151.2	0
7	T_{2g}	145.8	0	7	T_{1u}	160.2	32
8	T_{2g}	145.8	0	8	T_{1u}	160.2	32
9	T_{2g}	145.8	0	9	T_{1u}	160.2	32
10	E_g	196.0	0	10	E_g	223.6	0
11	E_g	196.0	0	11	E_g	223.6	0
12	A_{1g}	251.6	0	12	A_{1g}	260.7	0
13	T_{1u}	333.9	372	13	T_{1u}	404.3	371
14	T_{1u}	333.9	372	14	T_{1u}	404.3	371
15	T_{1u}	333.9	372	15	T_{1u}	404.3	371

SCS-MP2/def2-TZVPP-Cosmo (ε_r = 10)				SCS-MP2/def2-TZVPP-Cosmo (ε_r = 100)			
Nr.	Symmetry	Wavenumber / cm^{-1}	IR intensity / km mol^{-1}	Nr.	Symmetry	Wavenumber / cm^{-1}	IR intensity / km mol^{-1}
1	T_{2u}	113.8	0	1	T_{2u}	114.9	0
2	T_{2u}	113.8	0	2	T_{2u}	114.9	0
3	T_{2u}	113.8	0	3	T_{2u}	114.9	0
4	T_{2g}	155.6	0	4	T_{2g}	156.4	0
5	T_{2g}	155.6	0	5	T_{2g}	156.4	0
6	T_{2g}	155.6	0	6	T_{2g}	156.4	0
7	T_{1u}	167.8	62	7	T_{1u}	166.4	75
8	T_{1u}	167.8	62	8	T_{1u}	166.4	75
9	T_{1u}	167.8	62	9	T_{1u}	166.4	75
10	E_g	243.3	0	10	E_g	246.1	0
11	E_g	243.3	0	11	E_g	246.1	0
12	A_{1g}	280.7	0	12	A_{1g}	283.4	0
13	T_{1u}	392.0	875	13	T_{1u}	396.5	948
14	T_{1u}	392.0	875	14	T_{1u}	396.5	948
15	T_{1u}	392.0	875	15	T_{1u}	396.5	948

I) Coordinates of Optimized Structures

Cl_2

B3LYP

Cl	0.000000	0.000000	–1.007079
Cl	0.000000	0.000000	1.007079

MP2

Cl	0.000000	0.000000	–0.998704
Cl	0.000000	0.000000	0.998704

MP2 Cosmo (ε_r = 10)

Cl	0.000000	0.000000	–0.999952
Cl	0.000000	0.000000	0.999952

MP2 Cosmo (ε_r = 100)

Cl	0.000000	0.000000	–1.000157
Cl	0.000000	0.000000	1.000157

$[Cl_3]^–$

B3LYP

Cl	0.000000	0.000000	0.000000
Cl	0.000000	0.000000	2.349427
Cl	0.000000	0.000000	–2.349427

MP2

Cl	0.000000	0.000000	0.000000
Cl	0.000000	0.000000	2.309542
Cl	0.000000	0.000000	–2.309542

MP2 Cosmo (ε_r = 10)

Cl	0.000000	0.000000	0.000000
Cl	0.000000	0.000000	2.298025
Cl	0.000000	0.000000	–2.298025

MP2 Cosmo (ε_r = 100)

Cl	0.000000	0.000000	0.000000
Cl	0.000000	0.000000	2.296380
Cl	0.000000	0.000000	–2.296380

$[Cl(Cl_2)_2]^-$

B3LYP

Cl	0.222258	0.514373	–0.000000
Cl	2.678007	–0.057822	0.000000
Cl	4.790828	–0.624876	–0.000000
Cl	–0.148794	3.008114	–0.000000
Cl	–0.542299	5.160211	0.000000

MP2

Cl	0.165632	0.462139	0.000000
Cl	2.635412	–0.027498	–0.000001
Cl	4.725038	–0.481047	0.000000
Cl	–0.121860	2.963389	0.000001
Cl	–0.404222	5.083018	–0.000000

MP2 Cosmo (ε_r = 10)

Cl	–0.170316	0.150464	0.000004
Cl	2.554754	0.053896	–0.000022
Cl	4.613609	–0.012314	0.000013
Cl	–0.048356	2.875282	0.000016
Cl	0.050309	4.932672	–0.000010

MP2 Cosmo (ε_r = 100)

Cl	–0.250981	0.075830	0.000008
Cl	2.557894	0.069397	–0.000049
Cl	4.600761	0.065456	0.000029
Cl	–0.033255	2.876317	0.000032
Cl	0.125582	4.912999	–0.000021

S_8

B3LYP

S	2.189522	–0.906930	0.497590
S	2.189522	0.906930	–0.497590
S	0.906930	–2.189522	–0.497590
S	0.906930	2.189522	0.497590
S	–0.906930	–2.189522	0.497590
S	–0.906930	2.189522	–0.497590
S	–2.189522	–0.906930	–0.497590
S	–2.189522	0.906930	0.497590

MP2

S	2.162388	–0.895691	0.502364
S	2.162388	0.895691	–0.502364
S	0.895691	–2.162388	–0.502364
S	0.895691	2.162388	0.502364
S	–0.895691	–2.162388	0.502364
S	–0.895691	2.162388	–0.502364
S	–2.162388	–0.895691	–0.502364
S	–2.162388	0.895691	0.502364

MP2 Cosmo (ε_r = 10)

S	2.162390	–0.895691	0.503380
S	2.162390	0.895691	–0.503380
S	0.895691	–2.162390	–0.503380
S	0.895691	2.162390	0.503380
S	–0.895691	–2.162390	0.503380
S	–0.895691	2.162390	–0.503380
S	–2.162390	–0.895691	–0.503380
S	–2.162390	0.895691	0.503380

MP2 Cosmo (ε_r = 100)

S	2.162342	–0.895671	0.503551
S	2.162342	0.895671	–0.503551
S	0.895671	–2.162342	–0.503551
S	0.895671	2.162342	0.503551
S	–0.895671	–2.162342	0.503551
S	–0.895671	2.162342	–0.503551
S	–2.162342	–0.895671	–0.503551
S	–2.162342	0.895671	0.503551

S_2Cl_2

B3LYP

S	–0.973840	0.049395	–0.723364
S	0.973840	–0.049395	–0.723364
Cl	–1.581697	1.439759	0.723364
Cl	1.581697	–1.439759	0.723364

MP2

S	–0.979500	0.039295	–0.723227
S	0.979500	–0.039295	–0.723227
Cl	–1.527604	1.397023	0.723227
Cl	1.527604	–1.397023	0.723227

MP2 Cosmo (ε_r = 10)

S	–0.979202	0.035669	–0.727768
S	0.979202	–0.035669	–0.727768
Cl	–1.522878	1.395018	0.727768
Cl	1.522878	–1.395018	0.727768

MP2 Cosmo (ε_r = 100)

S	–0.979013	0.035042	–0.728724
S	0.979013	–0.035042	–0.728724
Cl	–1.522057	1.394663	0.728724
Cl	1.522057	–1.394663	0.728724

SCl_4

B3LYP

S	0.000000	0.000000	0.426710
Cl	0.000000	–1.600417	–0.824394
Cl	2.269667	0.000000	0.611040
Cl	–2.269667	0.000000	0.611040
Cl	0.000000	1.600417	–0.824394

MP2

S	0.000000	0.000000	0.453610
Cl	0.000000	–1.582369	–0.777184
Cl	2.240474	0.000000	0.550379
Cl	–2.240474	0.000000	0.550379
Cl	0.000000	1.582369	–0.777184

MP2 Cosmo (ε_r = 10)

S	0.000000	0.000000	0.448367
Cl	0.000000	–1.574174	–0.781131
Cl	2.259939	0.000000	0.556948
Cl	–2.259939	0.000000	0.556948
Cl	0.000000	1.574174	–0.781131

MP2 Cosmo (ε_r = 100)

S	0.000000	0.000000	0.447113
Cl	0.000000	–1.572774	–0.781965
Cl	2.263459	0.000000	0.558408
Cl	–2.263459	0.000000	0.558408
Cl	0.000000	1.572774	–0.781965

$[S_2Cl_3]^-$

B3LYP

S	–6.555245	–0.434200	0.687801
S	–4.629036	–0.659330	0.570898
Cl	–4.515906	–2.502738	–1.012970
Cl	–3.910196	1.027691	2.169884
Cl	–7.238507	1.005480	–0.778965

MP2

S	–6.530229	–0.449748	0.700441
S	–4.583744	–0.664399	0.567514
Cl	–4.598919	–2.436097	–1.009244
Cl	–4.003520	1.033949	2.119195
Cl	–7.132477	0.953199	–0.741257

MP2 Cosmo (ε_r = 10)

S	–6.559995	–0.474354	0.686745
S	–4.639647	–0.802338	0.450173
Cl	–4.628442	–2.377471	–1.008932
Cl	–3.909291	1.196895	2.281446
Cl	–7.111517	0.894171	–0.772783

MP2 Cosmo (ε_r = 100)

S	–6.575547	–0.490345	0.671065
S	–4.664618	–0.851051	0.413509
Cl	–4.636801	–2.388835	–1.028633
Cl	–3.858907	1.278334	2.359681
Cl	–7.113018	0.888800	–0.778974

$[SCl_3]^−$

B3LYP

S	0.000000	0.000000	-0.398814
Cl	2.369318	-0.000000	-0.637646
Cl	-2.369318	-0.000000	-0.637646
Cl	0.000000	-0.000000	1.675760

MP2

S	-0.000000	-0.000041	-0.438694
Cl	2.333946	0.000019	-0.587457
Cl	-2.333946	0.000019	-0.587457
Cl	0.000000	0.000003	1.615261

MP2 Cosmo (ε_r = 10)

S	-0.000026	0.000011	-0.448049
Cl	2.330705	-0.000002	-0.574770
Cl	-2.330675	-0.000002	-0.574764
Cl	-0.000004	-0.000006	1.599237

MP2 Cosmo (ε_r = 100)

Cl	-6.546107	-0.479562	0.684356
S	-4.561126	-0.833001	0.411389
Cl	-4.692983	-2.360457	-0.993016
Cl	-3.935656	1.221122	2.312893

$[SCl_5]^-$

B3LYP

S	0.000000	0.000000	–0.241552
Cl	1.647899	1.647899	–0.394026
Cl	0.000000	0.000000	1.817658
Cl	1.647899	–1.647899	–0.394026
Cl	–1.647899	1.647899	–0.394026
Cl	–1.647899	–1.647899	–0.394026

MP2

S	0.000000	0.000000	–0.273363
Cl	1.627010	1.627010	–0.371192
Cl	0.000000	0.000000	1.758131
Cl	1.627010	–1.627010	–0.371192
Cl	–1.627010	1.627010	–0.371192
Cl	–1.627010	–1.627010	–0.371192

MP2 Cosmo (ε_r = 10)

S	0.000000	0.000000	–0.269513
Cl	1.626260	1.626260	–0.370587
Cl	0.000000	0.000000	1.751861
Cl	1.626260	–1.626260	–0.370587
Cl	–1.626260	1.626260	–0.370587
Cl	–1.626260	–1.626260	–0.370587

MP2 Cosmo (ε_r = 100)

S	0.000000	0.000000	–0.268077
Cl	1.626210	1.626210	–0.370823
Cl	0.000000	0.000000	1.751369
Cl	1.626210	–1.626210	–0.370823
Cl	–1.626210	1.626210	–0.370823
Cl	–1.626210	–1.626210	–0.370823

$[SCl_6]^{2–}$

B3LYP

S	0.000000	0.000000	0.000000
Cl	0.000000	2.381674	0.000000
Cl	0.000000	0.000000	2.381674
Cl	2.381674	0.000000	0.000000
Cl	–2.381674	0.000000	0.000000
Cl	0.000000	–2.381674	0.000000
Cl	0.000000	0.000000	–2.381674

MP2

S	0.000000	0.000000	0.000000
Cl	0.000000	2.346834	0.000000
Cl	0.000000	0.000000	2.346834
Cl	2.346834	0.000000	0.000000
Cl	–2.346834	0.000000	0.000000
Cl	0.000000	–2.346834	0.000000
Cl	0.000000	0.000000	–2.346834

MP2 Cosmo (ε_r = 10)

S	0.000000	0.000000	0.000000
Cl	0.000000	2.317528	0.000000
Cl	0.000000	0.000000	2.317528
Cl	2.317528	0.000000	0.000000
Cl	–2.317528	0.000000	0.000000
Cl	0.000000	–2.317528	0.000000
Cl	0.000000	0.000000	–2.317528

MP2 Cosmo (ε_r = 100)

S	0.000000	0.000000	0.000000
Cl	0.000000	2.313361	0.000000
Cl	0.000000	0.000000	2.313361
Cl	2.313361	0.000000	0.000000
Cl	–2.313361	0.000000	0.000000
Cl	0.000000	–2.313361	0.000000
Cl	0.000000	0.000000	–2.313361

m) References

[1] P. Voßnacker, S. Steinhauer, J. Bader, S. Riedel, *Chem. Eur. J.* **2020**, *26*, 13256.
[2] G. M. Sheldrick, *Acta Cryst.* **2008**, *A64*, 112.
[3] G. M. Sheldrick, *Acta Cryst. C* **2015**, *71*, 3.
[4] O. V. Dolomanov, L. J. Bourhis, R. J. Gildea, J. A. K. Howard, H. Puschmann, *J. Appl. Cryst.* **2009**, *42*, 339.
[5] A. Klamt, G. Schüürmann, *J. Chem. Soc., Perkin Trans. 2* **1993**, 799.
[6] TURBOMOLE GmbH, *TURBOMOLE V7.3. a development of University of Karlsruhe and Forschungszentrum Karlsruhe*, **2018**.
[7] a) F. Neese, *WIREs Comput. Mol. Sci.* **2012**, *2*, 73; b) F. Neese, *WIREs Comput. Mol. Sci.* **2017**, *2*, e1327.
[8] E. D. Glendening, J. K. Badenhoop, A. E. Reed, J. E. Carpenter, J. A. Bohmann, C. M. Morales, P. Karafiloglou, C. R. Landis, F. Weinhold, *NBO 7.0*, Theoretical Chemistry Institute, University of Wisconsin, Madison, WI, **2018**.
[9] M. J. Frisch, G. W. Trucks, H. B. Schlegel, G. E. Scuseria, M. A. Robb, J. R. Cheeseman, G. Scalmani, V. Barone, G. A. Petersson, H. Nakatsuji, X. Li, M. Caricato, A. V. Marenich, J. Bloino, B. G. Janesko, R. Gomperts, B. Mennucci, H. P. Hratchian, J. V. Ortiz, A. F. Izmaylov, J. L. Sonnenberg, D. Williams-Young, F. Ding, F. Lipparini, F. Egidi, J. Goings, B. Peng, A. Petrone, T. Henderson, D. Ranasinghe, V. G. Zakrzewski, J. Gao, N. Rega, G. Zheng, W. Liang, M. Hada, M. Ehara, K. Toyota, R. Fukuda, J. Hasegawa, M. Ishida, T. Nakajima, Y. Honda, O. Kitao, H. Nakai, T. Vreven, K. Throssell, J. A. Montgomery, Jr., J. E. Peralta, F. Ogliaro, M. J. Bearpark, J. J. Heyd, E. N. Brothers, K. N. Kudin, V. N. Staroverov, T. A. Keith, R. Kobayashi, J. Normand, K. Raghavachari, A. P. Rendell, J. C. Burant, S. S. Iyengar, J. Tomasi, M. Cossi, J. M. Millam, M. Klene, C. Adamo, R. Cammi, J. W. Ochterski, R. L. Martin, K. Morokuma, O. Farkas, J. B. Foresman, and D. J. Fox, *Gaussian 16*, Gaussian, Inc., Wallingford CT, **2016**.
[10] a) A. D. Becke, *J. Chem. Phys.* **1993**, *98*, 5648; b) C. Lee, W. Yang, R. G. Parr, *Phys. Rev. B* **1988**, *37*, 785; c) S. H. Vosko, L. Wilk, M. Nusair, *Can. J. Phys.* **1980**, *58*, 1200; d) P. J. Stephens, F. J. Devlin, C. F. Chabalowski, M. J. Frisch, *J. Phys. Chem.* **1994**, *98*, 11623; e) A. D. Becke, E. R. Johnson, *J. Chem. Phys.* **2005**, *123*, 154101; f) E. R. Johnson, A. D. Becke, *J. Chem. Phys.* **2005**, *123*, 24101; g) E. R. Johnson, A. D. Becke, *J. Chem. Phys.* **2006**, *124*, 174104; h) E. Caldeweyher, S. Ehlert, A. Hansen, H. Neugebauer, S. Spicher, C. Bannwarth, S. Grimme, *J. Chem. Phys.* **2019**, *150*, 154122.
[11] S. Grimme, J. Antony, S. Ehrlich, H. Krieg, *J. Chem. Phys.* **2010**, *132*, 154104.
[12] S. Grimme, *J. Chem. Phys.* **2003**, *118*, 9095.
[13] F. Weigend, R. Ahlrichs, *Phys. Chem. Chem. Phys.* **2005**, *7*, 3297.
[14] F. Weigend, A. Köhn, C. Hättig, *J. Chem. Phys.* **2002**, *116*, 3175.
[15] P. R. Spackman, M. J. Turner, J. J. McKinnon, S. K. Wolff, D. J. Grimwood, D. Jayatilaka, M. A. Spackman, *J. Appl. Cryst.* **2021**, *54*, 1006.
[16] a) D. E. Woon, T. H. Dunning, *J. Chem. Phys.* **1993**, *98*, 1358; b) T. H. Dunning, *J. Chem. Phys.* **1989**, *90*, 1007.
[17] R. Dovesi, A. Erba, R. Orlando, C. M. Zicovich-Wilson, B. Civalleri, L. Maschio, M. Rérat, S. Casassa, J. Baima, S. Salustro et al., *WIREs Comput Mol Sci* **2018**, *8*, e1360.
[18] S. Grimme, *J. Comput. Chem.* **2006**, *27*, 1787.
[19] C. Gatti, *Acta Cryst. A* **1996**, *52*, C555-C556.
